# scRADAR: Dissecting intratumoral drug response heterogeneity at single-cell resolution via mechanism-guided prototype routing

**DOI:** 10.1371/journal.pcbi.1014392

**Published:** 2026-06-26

**Authors:** Ren Qi, Wenjie Teng, Xin Yang, Peng Han, Alexey K. Shaytan, Bin Liu

**Affiliations:** 1 School of Computer Science and Technology, Beijing Institute of Technology, Beijing, China; 2 Zhongguancun Academy, Beijing, China; 3 Department of Biology, Lomonosov Moscow State University, Moscow, Russia; 4 SMBU-MSU-BIT Joint Laboratory on Bioinformatics and Engineering Biology, Shenzhen MSU-BIT University, Shenzhen, Guangdong, China; Shanghai Institute of Nutrition and Health, Chinese Academy of Sciences, CHINA

## Abstract

Precision oncology requires resolving intratumoral heterogeneity to identify drug-resistant cell states associated with treatment failure and relapse. Although single-cell RNA sequencing enables characterization of heterogeneous resistance-associated states, single-cell drug-response phenotype prediction remains challenging because of sparsity, noise, class imbalance, and limited mechanistic interpretability. Here, we present scRADAR (Response Analysis via Drug-Aware Routing), a mechanism-guided prototype routing framework for predicting and interpreting drug-response phenotypes at single-cell resolution. Rather than relying on cell-line–anchored transfer learning, scRADAR learns directly from labeled single-cell cohorts. The framework integrates metabolic and signaling pathway activities to form a dual-view cellular representation, conditions pathway embeddings on drug mechanisms through feature-wise linear modulation, and uses sparse prototype routing to decompose predictions into interpretable response archetypes. Across nine independent cohorts, scRADAR showed strong predictive performance and consistent cross-cohort behavior, particularly under imbalanced settings. Post hoc attribution analyses highlighted candidate TGF-β-associated epithelial-to-mesenchymal transition signatures in Erlotinib-associated Resistant-labeled states and cytoskeletal/metabolic response-associated signatures in BET-inhibitor-associated Resistant-labeled states. These results suggest that scRADAR provides an interpretable framework for single-cell drug-response phenotype prediction and for generating hypotheses about resistance-associated programs from heterogeneous tumor transcriptomes.

## Author summary

Tumors are made up of diverse cell populations, and some of these cells can survive therapy and later drive relapse. Single-cell RNA sequencing can reveal this hidden diversity, but turning these data into reliable and interpretable drug-response phenotype predictions remains difficult. In this study, we developed scRADAR, a computational framework that predicts cell-level drug-response phenotypes while helping interpret why cells are assigned to Sensitive or Resistant response-associated labels. Instead of treating prediction as a black box, we designed scRADAR to connect cellular pathway activity with drug mechanism and to organize cells into a small number of interpretable latent response archetypes. Across nine independent datasets, scRADAR showed strong predictive performance and remained informative in challenging settings such as imbalanced cohorts. It also highlighted candidate response-associated pathway signatures, including TGF-β-related signals in Erlotinib-associated Resistant-labeled states and cytoskeletal/metabolic signatures in BET-inhibitor-associated Resistant-labeled states.

## 1. Introduction

Precision oncology fundamentally relies on accurately predicting therapeutic responses to tailor treatments for individual patients [1]. However, tumors are not uniform entities but complex ecosystems characterized by profound intratumoral heterogeneity (ITH) [2–4]. Traditional bulk RNA sequencing, which averages gene expression across millions of cells, inevitably masks clinically relevant resistance-associated cell states that can contribute to therapeutic failure and tumor relapse [5–7]. In contrast, single-cell RNA sequencing (scRNA-seq) has emerged as a transformative technology, offering the resolution required to dissect cellular heterogeneity and identify cell states associated with cohort-harmonized response-associated labels [8–13]. Developing computational models capable of predicting drug-response phenotypes at the single-cell level is therefore important for prioritizing candidate resistance-associated programs and supporting hypothesis generation [8,11,14,15] and response stratification in precision oncology research [1,16].

In recent years, deep learning has demonstrated remarkable potential in deciphering scRNA-seq profiles [17–22]. Computational pharmacogenomics has traditionally relied on bulk transcriptomic data to model dose–response relationships [13]. However, only limited efforts have extended these models to the single-cell level to account for intratumoral heterogeneity [23]. To address the scarcity of labeled single-cell drug-response phenotype data, pioneering approaches such as scDEAL [23], scAdaDrug [24], and recent frameworks like scDrug and scDrug+ [25] have employed domain adaptation and transfer learning. These methods typically aim to bridge the gap between large-scale cell-line resources (*e.g.*, GDSC [26], CCLE [27]) and clinical single-cell samples [28]. Subsequent methods like SSDA4Drug [29] have further introduced semi-supervised frameworks to leverage unlabeled cells, while SCAD [30] and scATD [31] incorporate attention mechanisms to capture long-range gene dependencies. A critical caveat persists in current approaches: despite being designed for single-cell applications, most models are predominantly trained on bulk RNA-seq data, while single-cell datasets are largely confined to inference or limited fine-tuning.

Despite these advances, bulk-to-single transfer can introduce important limitations for modeling drug-response phenotypes at single-cell resolution. Because bulk sequencing averages expression across large cell populations, it may underrepresent clinically relevant resistance-associated cell states and thereby attenuate the very heterogeneity that single-cell models aim to resolve. Beyond this conceptual mismatch, several methodological challenges remain. First, representation learning is often vulnerable to sparsity and noise, particularly when latent spaces are derived directly from gene-level expression and therefore retain substantial dropout-driven variance and technical artifacts [32–34]. Second, explicit pharmacological constraints are not always incorporated into single-cell drug-response phenotype models, even though therapeutic response is shaped by target engagement and pathway context [35–38]. Third, mechanistic interpretability remains limited in many current frameworks, which often output scalar response scores without a transparent link to the biological programs underlying the prediction [29,30,39]. These considerations motivate the development of frameworks that operate directly on single-cell data while integrating pharmacological priors and preserving biological interpretability. For clinical utility, such models should also provide traceable explanations—such as response-relevant pathways [4,40] or representative prototypes [41,42]—to connect predictions with actionable biological hypotheses [43].

**Fig 1:**
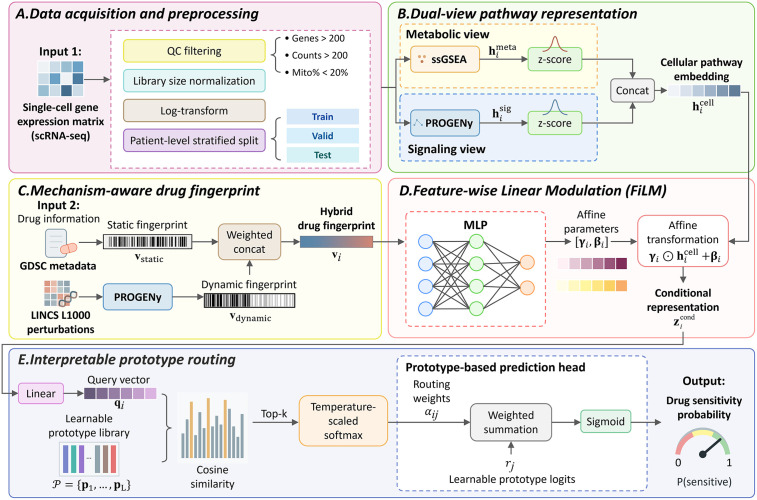
Overview of the scRADAR framework. **(A)** scRNA-seq data are QC-filtered, library-size normalized, log-transformed, and split with patient-level stratification. **(B)** Dual-view pathway encoding concatenates ssGSEA (metabolic) and PROGENy (signaling) scores into a unified cellular embedding. **(C)** Mechanism-aware drug fingerprints fuse static GDSC descriptors with dynamic LINCS L1000 perturbation signatures. **(D)** FiLM conditions cellular embeddings via drug-derived affine parameters to generate a mechanism-conditional representation. **(E)** Prototype routing computes cosine similarities to learnable prototypes, applies sparse top-*k* selection, and outputs a predicted response probability via similarity-weighted aggregation.

To address these challenges, we propose scRADAR (Response Analysis via Drug-Aware Routing), a mechanism-guided prototype network designed to predict and dissect distinct drug-response phenotypes within the noisy single-cell landscape (Fig 1). The framework incorporates three key innovations to bridge the gap between predictive accuracy and mechanistic interpretability: (1) a high-fidelity dual-view representation that integrates metabolic and signaling pathway activities to mitigate scRNA-seq noise; (2) mechanism-guided modulation, which conditions cellular embeddings on hybrid drug fingerprints using Feature-wise Linear Modulation (FiLM) to introduce drug-dependent pharmacological conditioning; and (3) interpretable prototype routing, which decomposes complex predictions into sparse, biologically meaningful archetypes, transforming black-box scores into a transparent decision process. By synergizing these components, scRADAR not only denoises single-cell inputs but also facilitates post hoc interpretation of mechanism-associated response patterns. Across datasets, scRADAR demonstrated strong predictive performance in drug-response phenotype prediction relative to representative transfer-learning baselines and yielded model-derived post hoc interpretations that connect predictions to candidate biological programs rather than opaque scores. Together, these results suggest that scRADAR can help characterize heterogeneous response phenotypes and organize Resistant-labeled states into interpretable, post hoc response-associated patterns.

## 2. Results

### 2.1. scRADAR establishes a high-performance benchmark across diverse single-cell drug-response phenotype cohorts

**Fig 2:**
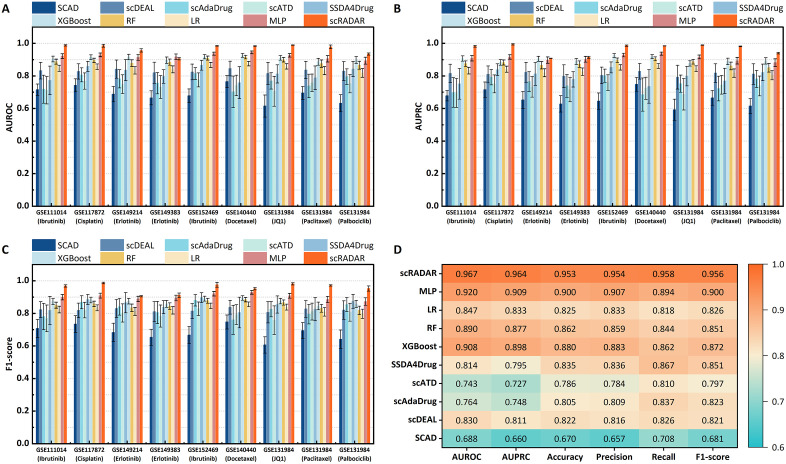
scRADAR achieves superior predictive performance and stability across nine held-out scRNA-seq drug-response phenotype cohorts. **(A–C)** Bar plots comparing the mean AUROC **(A)**, AUPRC **(B)**, and F1-score **(C)** of scRADAR with nine baseline methods, including representative transfer-learning and target-domain supervised comparators, across the nine held-out test cohorts. Error bars denote 95% t-intervals derived from cross-validation-based models, indicating robustness to training data variations. **(D)** Cohort-averaged performance heat map summarizing AUROC, AUPRC, Accuracy, Precision, Recall, and F1-score across all methods; warmer colors indicate better performance.

We systematically evaluated scRADAR on nine held-out scRNA-seq drug-response phenotype cohorts using a unified benchmark that included five representative bulk-to-single transfer-learning methods (SCAD [30], scATD [31], scDEAL [23], SSDA4Drug [29], and scAdaDrug [24]), a scGEN-adapted single-cell perturbation-oriented baseline, and four conventional supervised classifiers trained on the same target-domain single-cell splits (LR [44], RF [45], MLP [46], and XGBoost [47]). The nine baseline methods shown in Fig 2 include the five transfer-learning baselines and four target-domain supervised classifiers, whereas the scGEN-adapted comparator is reported separately in S5 Table. Across this unified evaluation framework, scRADAR showed the strongest overall performance profile, achieving cohort-averaged AUROC, AUPRC, Accuracy, Precision, Recall, and F1-score values of 0.967, 0.964, 0.953, 0.954, 0.958, and 0.956, respectively. These results suggest that the performance of scRADAR reflects not only access to labeled target-domain cells but also the contribution of its mechanism-guided representation learning and prototype-routing design.

We first assessed global discrimination using AUROC (Fig 2A). Among the transfer-learning baselines, scDEAL provided the strongest average ranking performance with a mean AUROC of 0.830, whereas MLP was the most competitive supervised comparator with a mean AUROC of 0.920. Against these reference points, scRADAR improved the mean AUROC by 0.137 and 0.047, corresponding to relative gains of 16.5% and 5.1%, respectively. Notably, scRADAR maintained uniformly high cohort-level discrimination, with all nine AUROC values exceeding 0.90 and spanning a narrow high-performance range from 0.905 to 0.989. The magnitude of the improvement was especially evident in several representative cohorts. In GSE111014 (Ibrutinib), AUROC increased from 0.921 for MLP to 0.987 for scRADAR, an absolute gain of 0.066 (+7.2%). Likewise, in GSE131984 (Paclitaxel), scRADAR reached an AUROC of 0.978, outperforming MLP by 0.073 (+8.1%). GSE140440 (Docetaxel) represents a particularly challenging cohort because it is the smallest dataset in the benchmark after preprocessing (n_total_ = 284) and contains two prostate cancer cell-line backgrounds, DU145 and PC3. In this setting, the held-out evaluation is more sensitive to sampling variation, class-boundary instability, and cell-line-specific transcriptional differences. Even under this small-sample and mixed-background setting, scRADAR sustained a strong margin, yielding an AUROC of 0.983 versus 0.944 for MLP.

We next turned to AUPRC and F1-score, which provide a more operational view of classification quality by jointly reflecting ranking behavior and thresholded decision performance (Fig 2B and 2C). Here, the advantage of scRADAR became even more pronounced. Its mean AUPRC reached 0.964, compared with 0.811 for the strongest transfer-learning baseline and 0.909 for the strongest supervised baseline, corresponding to absolute gains of 0.153 and 0.055. Expressed in relative terms, these improvements amount to 18.9% over scDEAL and 6.1% over MLP. An analogous pattern was observed for F1-score: scRADAR achieved a mean F1-score of 0.956, exceeding SSDA4Drug (0.851) by 0.105 (+12.3%) and MLP (0.900) by 0.056 (+6.2%). Importantly, these gains were not confined to isolated datasets. scRADAR produced consistently elevated cohort-level AUPRC values, all above 0.90, ranging from 0.908 to 0.993, while F1-scores remained uniformly high across the benchmark, ranging from 0.905 to 0.986. In GSE131984 (Paclitaxel), for example, AUPRC increased from 0.893 for MLP to 0.981 for scRADAR, a gain of 0.088 (+9.9%), while F1-score rose from 0.885 to 0.971, a gain of 0.086 (+9.7%). A similarly clear separation was observed in GSE111014 (Ibrutinib), where scRADAR improved AUPRC from 0.908 to 0.982 and F1-score from 0.899 to 0.968 relative to MLP.

The cohort-averaged heat map in Fig 2D further highlights the coordinated nature of these gains. Rather than improving one metric at the expense of another, scRADAR occupied the leading position across all six summary measures. Relative to the best overall supervised baseline, MLP, scRADAR increased Accuracy from 0.900 to 0.953, Precision from 0.907 to 0.954, Recall from 0.894 to 0.958, and F1-score from 0.900 to 0.956. These correspond to absolute improvements of 0.053, 0.047, 0.064, and 0.056, respectively, or relative gains of 5.9%, 5.2%, 7.2%, and 6.2%. Particularly noteworthy is the increase in Recall, suggesting that scRADAR more effectively preserves Sensitive/Resistant label separation at the final decision threshold while maintaining strong precision. This balanced improvement profile is difficult to obtain with conventional classifiers and is consistent with the view that biologically grounded pathway encoding and sparse prototype routing yield a more robust decision geometry than generic supervised mapping.

An additional point worth emphasizing is that the supervised baselines were themselves highly competitive. MLP reached mean AUROC and AUPRC values of 0.920 and 0.909, while XGBoost achieved 0.908 and 0.898, underscoring the stringency of the benchmark. Against this stronger comparator tier, scRADAR nevertheless maintained a consistent performance margin across the summary metrics. Taken together, these results position scRADAR as a robust and competitive framework within the unified benchmark and support the view that its performance is linked to the combination of biologically grounded pathway encoding, mechanism-aware conditioning, and prototype-based decision structure.

We further included a scGEN-adapted baseline to compare scRADAR with a representative single-cell perturbation-oriented model. Under the same target-domain split and threshold-selection protocol, the scGEN-adapted baseline achieved cohort-averaged AUROC, AUPRC, and F1-score values of 0.884, 0.867, and 0.852, respectively (S5 Table). These results indicate that a single-cell perturbation-style latent representation is informative for this task, but its performance remains below both the strongest target-domain supervised comparator and full scRADAR. Thus, the performance advantage of scRADAR cannot be attributed simply to using single-cell data, but is more consistent with the combined contribution of pathway-level encoding, mechanism-aware drug conditioning, and sparse prototype routing.

### 2.2. Systematic ablation disentangles component-wise contributions and validates architectural choices

To rigorously attribute performance gains to specific design choices, we conducted a comprehensive component-wise ablation study, with detailed performance metrics summarized in Table 1. Results are reported as mean values with 95% t-intervals to quantify both the magnitude of predictive power and the stability of model training.

**Table 1:** Ablation study of scRADAR components and input feature configurations. Data are reported as mean ±95% t-intervals derived from five fold-trained models (*K* = 5). The best performance in each metric is highlighted in bold. Combined indicates the full feature set (ssGSEA + PROGENy for pathways; Static + Dynamic for drugs). Abbreviations: FiLM, feature-wise linear modulation; Proto., prototype-based routing; Top-*k*, sparse routing using the *k* most similar prototypes. The “MLP head” variant replaces the prototype routing mechanism with a standard classifier.

Variant	Pathway features	Drug features	FiLM	Prototype routing	Top-*k*	AUROC	AUPRC	F1
**scRADAR (Ours)**	**ssGSEA + PROGENy**	**static + dynamic**	**✓**	**✓**	**✓**	**0.967 ± 0.005**	**0.964 ± 0.003**	**0.956 ± 0.007**
**Pathway view (single-view variants)**								
ssGSEA only	ssGSEA	static + dynamic	✓	✓	✓	0.943 ± 0.017	0.934 ± 0.017	0.929 ± 0.017
PROGENy only	PROGENy	static + dynamic	✓	✓	✓	0.937 ± 0.015	0.926 ± 0.014	0.923 ± 0.015
**Drug fingerprint (single-source variants)**								
Static fingerprint only	ssGSEA + PROGENy	static	✓	✓	✓	0.958 ± 0.008	0.950 ± 0.009	0.944 ± 0.008
Dynamic fingerprint only	ssGSEA + PROGENy	dynamic	✓	✓	✓	0.948 ± 0.013	0.938 ± 0.014	0.932 ± 0.013
**Architecture variants**								
No FiLM modulation	ssGSEA + PROGENy	static + dynamic	✗	✓	✓	0.962 ± 0.010	0.956 ± 0.009	0.950 ± 0.009
MLP head (no prototype routing)	ssGSEA + PROGENy	static + dynamic	✓	✗	✗	0.928 ± 0.017	0.915 ± 0.016	0.907 ± 0.018
Dense routing (no top-*k*)	ssGSEA + PROGENy	static + dynamic	✓	✓	✗	0.965 ± 0.006	0.960 ± 0.006	0.953 ± 0.007

We first examined the synergy of multi-view biological representations by isolating the metabolic (ssGSEA) and signaling (PROGENy) inputs. The integration of both views proved essential for robust discrimination, as evidenced by the performance deficits observed in single-view models. Specifically, relying solely on ssGSEA or PROGENy led to absolute AUROC decreases of 2.4% and 3.0%, respectively, relative to the full model’s score of 0.967. A similar trend was observed in AUPRC, where the full model (0.964) outperformed the single-view variants by approximately 3.0% to 3.8%. Notably, as detailed in the stability analysis, the uncertainty intervals for single-view models widened by over 3-fold (e.g., AUROC error margin increasing from ±0.5% to ±1.7%), indicating that dual-view integration not only enhances predictive accuracy but also stabilizes model convergence against training data variations. This confirms that metabolic state and signaling potential provide non-redundant information: one captures the energetic baseline, while the other reflects dynamic responsiveness. This interpretation was further reinforced by a direct cross-view correlation analysis. Specifically, we quantified pairwise Pearson correlations between ssGSEA-derived metabolic features and PROGENy-derived signaling features within each cohort and observed only modest overall coupling across datasets (S1 Fig). Across the nine cohorts, mean absolute cross-view correlation ranged from 0.052 to 0.178, whereas median absolute correlation ranged from 0.036 to 0.139, arguing against the interpretation that the two pathway views are simple re-encodings of the same signal. In a representative cohort, GSE152469, the compact cross-view matrix revealed a sparse pattern dominated by weak correlations, with only a limited subset of pathway pairs showing stronger local correspondence. Together, these results indicate that the two views are partially connected yet globally complementary.

We also assessed whether the main performance profile was sensitive to the feature-normalization strategy. Across the four pathway-feature normalization settings, full scRADAR remained within a narrow performance range (S2 Table). The default z-score setting achieved AUROC, AUPRC, and F1-score values of 0.967, 0.964, and 0.956, respectively, while the alternative normalization settings produced comparable values. These results indicate that the predictive conclusions were not driven by a single normalization choice.

To address the dependence of pathway-based modeling on the selected gene-set collection, we further performed pathway-view and pathway-set sensitivity analyses. The ssGSEA-only and PROGENy-only variants remained informative but were lower than the full dual-view representation, supporting the use of both pathway activity and signaling perturbation views (S3 Table). Replacing Reactome with Hallmark produced comparable performance within the same range, and the expanded Reactome + Hallmark + PROGENy representation yielded comparable ranking performance. These results indicate that the main conclusions were not driven by a single pathway collection, although disease- or lineage-specific gene sets may still improve interpretive resolution in specific cohorts.

Because B-cell malignancy pathways may be particularly relevant to the Ibrutinib cohort GSE111014, we additionally evaluated B-cell-specific signatures, including CD40 signaling, NF-κB signaling, B-cell receptor signaling, BTK downstream signaling, B-cell activation, apoptosis, antigen presentation, and proliferation-related programs. Adding these signatures produced comparable performance with small numerical changes in AUPRC and F1-score (S4 Table). The expanded B-cell setting retained the same B-cell signatures and further incorporated Hallmark features, again yielding performance within the same range. These results suggest that B-cell-specific signatures provide complementary context information, while the default Reactome + PROGENy representation already captures the major response-associated signal in this cohort.

We next examined the contribution of the hybrid drug fingerprint, which combines pharmacological priors with perturbation-derived context. Using static fingerprints alone already yielded a strong baseline, with AUROC and F1-score values of 0.958 and 0.944, respectively. Incorporating dynamic LINCS L1000 signatures further improved performance across all three summary metrics, raising AUPRC to 0.964 and F1-score to 0.956. Notably, the dynamic-only variant remained weaker than the static-only variant, trailing by 1.0% in AUROC and 1.2% in F1-score. This pattern suggests that perturbation-derived transcriptional information contributes useful context, but is most effective when anchored by canonical target and pathway annotations. Taken together, these comparisons support the value of integrating mechanism-based priors with data-driven perturbational signals rather than relying on either source alone.

Architectural ablations further clarified which components were most responsible for the performance of the full model. Among these variants, replacing prototype routing with a standard MLP head produced the largest degradation, with AUROC falling from 0.967 to 0.928, AUPRC from 0.964 to 0.915, and F1-score from 0.956 to 0.907. The magnitude of this drop indicates that the prototype-based decision structure is not merely an interpretability add-on, but a central component for representing heterogeneous response modes within and across cohorts. By contrast, removing FiLM modulation yielded a smaller yet consistent decline across metrics, including a 0.6% reduction in F1-score, indicating that FiLM-based drug-dependent pathway reweighting contributes complementary value beyond the pathway representation itself. Viewed together, these results support a division of labor within the architecture: FiLM provides drug-dependent pathway reweighting, whereas prototype routing organizes this conditioned information into interpretable response archetypes.

Finally, the comparison between sparse and dense routing highlights the value of explicit decision sparsity. Relative to dense routing, top-k sparse routing produced a modest but consistent gain in predictive performance while yielding a clearer improvement in robustness. In particular, the AUPRC uncertainty interval was reduced by 50%, from ±0.006 to ±0.003, indicating that sparse routing dampens prototype-level noise and stabilizes optimization under training variability. Importantly, this gain in stability was achieved without sacrificing predictive accuracy, suggesting that sparsity improves not only interpretability but also the reliability of the learned decision geometry.

Because the component-wise ablations above were performed under the primary within-cohort held-out-cell protocol, we further assessed whether the mechanism-aware design retained value when the test drug itself was unseen during model development. We performed an additional controlled within-study unseen-drug transfer analysis using the three drug-specific subsets of GSE131984. In each split, two drug-specific subsets were used for training and the remaining drug was held out for testing, with no cells from the held-out drug used for training, validation, threshold selection, or hyperparameter selection. This setting controls for study, platform, and cell-line background while testing transfer across transcriptionally distinct drug responses.

Compared with the cell-only MLP, drug-aware models improved macro-average performance, indicating that mechanism-aware drug information provided transferable signal across held-out drug responses (S6 Table). Full scRADAR achieved the highest macro-average AUROC, AUPRC, and F1-score, although drug-specific profiles varied across held-out drugs. These results support the use of FiLM-conditioned pathway reweighting and sparse prototype routing as a compact mechanism-aware interaction design, without implying that the model captures all higher-order context-specific drug-cell interactions.

### 2.3. Hybrid drug fingerprints show consistency with canonical mechanisms and highlight candidate context-specific response-associated features

**Fig 3:**
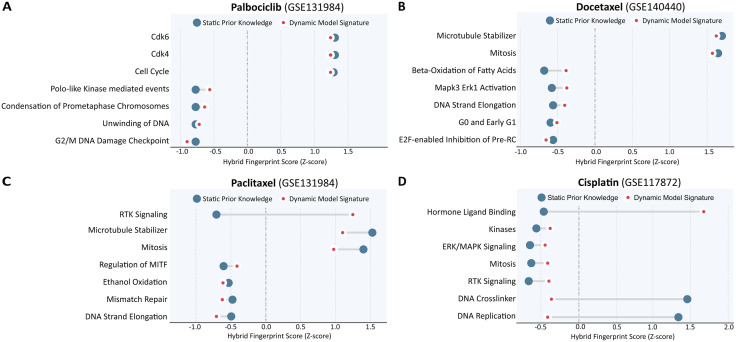
Hybrid drug fingerprints show canonical and context-specific score patterns. **(A–D)** Drug-fingerprint score profiles for four representative drugs. The x-axis shows the z-scored hybrid drug-fingerprint score. Blue points denote static prior-derived mechanism scores, whereas red points denote dynamic perturbation-derived pathway scores. Static and dynamic scores were standardized separately within each drug-specific profile. These scores are intended as post hoc diagnostic summaries rather than direct measurements of pathway activation.

To examine whether the learned drug representations were consistent with pharmacological knowledge, we analyzed post hoc drug-fingerprint score profiles using the procedure described in Section 4.11.1, with the results shown in Fig 3. By comparing static prior-derived mechanism scores with dynamic perturbation-derived pathway scores, we examined whether the diagnostic score profiles were consistent with known mechanisms of action.

For highly specific targeted therapies, the model showed score patterns consistent with known mechanisms, illustrated in Fig 3A–C. In the case of Palbociclib (a CDK4/6 inhibitor), Fig 3A shows that the model assigned top-ranking weights to Cdk4, Cdk6, and Cell Cycle programs in both static and dynamic views, supporting the interpretation that cell-cycle-related features were top-ranked in the Palbociclib diagnostic score profile. Similarly, the analyses for microtubule-stabilizing agents, such as Docetaxel (Fig 3B) and Paclitaxel (Fig 3C), consistently prioritized Microtubule Stabilizer and Mitosis signatures. This concordance between prior knowledge and dynamic perturbation-derived score profiles supports the interpretation that scRADAR uses pharmacologically relevant features in these representative cases.

Beyond recapitulating known pharmacological associations, the dynamic component highlighted candidate non-canonical associations that were not represented by static priors alone, as exemplified by the Cisplatin cohort in Fig 3D. While the static prior correctly highlighted DNA Replication and DNA Crosslinker terms, the data-driven dynamic signature unexpectedly prioritized Hormone Ligand Binding as a top-ranked feature, shifting it from a negative static score to a highly positive dynamic weight. We interpret this pattern as a candidate context-specific resistance-associated signal, whereby hormone-related signaling may mark a cohort-specific transcriptional association with Cisplatin response labels in this retrospective dataset. Although this interpretation remains hypothesis-generating, it is consistent with independent studies reporting associations between steroid hormone signaling and platinum-response phenotypes in reproductive cancers [48–51]. We therefore interpret these dynamic signatures as model-derived diagnostic signals that may prioritize candidate context-specific hypotheses, rather than as direct evidence of pathway activation, inhibition, or causal resistance mechanisms.

### 2.4. Response landscapes reveal intratumoral heterogeneity and motivate calibration beyond a single operating point

**Fig 4:**
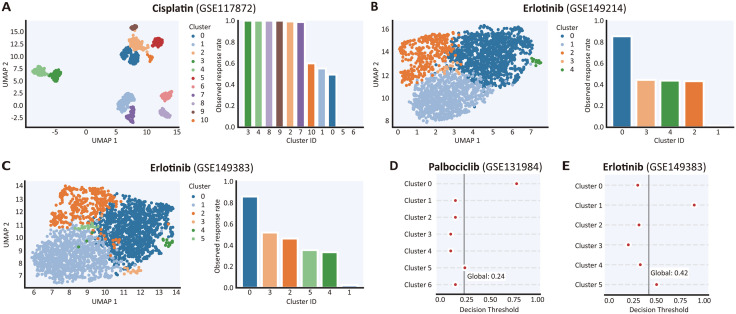
Intratumoral heterogeneity motivates calibration beyond a single operating point. **(A–C)** Left: UMAP embeddings of tumor cells colored by transcriptional clusters. Right: Bar plots of the fraction of cells assigned to the Sensitive label within each cluster, revealing pronounced within-cohort variability. **(D–E)** Threshold calibration for Palbociclib and Erlotinib. The vertical grey line denotes the global threshold selected on the validation set. Red points show post hoc cluster-wise F1-optimizing thresholds computed using cluster-specific response-associated labels after model evaluation; these thresholds were used only for descriptive calibration analysis. The difference between global and cluster-wise thresholds summarizes local score-label calibration heterogeneity rather than a prospective threshold-adaptation procedure.

A critical challenge in precision oncology is resolving clinically relevant intratumoral heterogeneity (ITH), where resistance-associated subpopulations can influence cohort-level response patterns. To assess scRADAR’s resolution, we projected single-cell predictions onto low-dimensional manifolds and examined how fractions of cells assigned to the Sensitive label vary across transcriptional neighborhoods, as illustrated in Fig 4.

Transcriptional clustering delineates discrete phenotypic neighborhoods, within which scRADAR reveals sharply divergent response profiles (Fig 4A–C). In the Cisplatin cohort, visualized in Fig 4A, specific subpopulations, including Clusters 3, 4, 8, and 9, exhibited near-uniform enrichment of the Sensitive label, with observed Sensitive-label fractions approaching 1.0. In contrast, distinct groups such as Clusters 5 and 6 show near-zero Sensitive-label fraction, indicating near-uniform enrichment of the Resistant label, with additional neighborhoods occupying intermediate states. A similar structure is observed in Erlotinib-treated cohorts (Fig 4B and 4C), where a neighborhood enriched for the Sensitive label, specifically Cluster 0 with a Sensitive-label fraction of approximately 0.85, coexists with a Resistant-enriched neighborhood, Cluster 1, with Sensitive-label fractions near zero in both datasets. These patterns are consistent with non-random organization of response-associated labels across transcriptional neighborhoods, although the UMAP visualization itself should be interpreted as descriptive rather than as direct evidence of causal biological structure. To statistically support this descriptive observation, we performed cluster-level response-label enrichment tests using label permutation while preserving cluster sizes and the cohort-level label composition (S7 Table). In the Cisplatin cohort, multiple clusters showed significant enrichment for either Sensitive- or Resistant-labeled cells after FDR correction, including Sensitive-enriched Clusters 2, 3, 4, 7, 8, and 9 and Resistant-enriched Clusters 0, 1, 5, and 6. In the two Erlotinib cohorts, the largest transcriptional neighborhoods showed reproducible label-enrichment patterns, with Cluster 0 enriched for Sensitive-labeled cells and Cluster 1 enriched for Resistant-labeled cells in both datasets. Smaller clusters were interpreted cautiously because their enrichment estimates were less stable.

This pronounced heterogeneity challenges the assumption that a single operating point is sufficient across all subpopulations, as analyzed in Fig 4D and 4E. Using validation-guided calibration, we selected global thresholds (vertical grey lines), specifically 0.24 for Palbociclib and 0.42 for Erlotinib, to optimize cohort-level performance. While these global operating points promote generalizability, post hoc cluster-wise analyses, computed as described in Section 4.11.2 and shown as red points, revealed substantial deviations in local score-label calibration patterns. Fig 4D demonstrates that in the Palbociclib cohort, Cluster 0 favors a markedly higher threshold compared to the global operating point, whereas the majority of other clusters favor lower thresholds. We further characterized Palbociclib Cluster 0 to clarify why this neighborhood showed a markedly higher post hoc threshold. Cluster 0 was Resistant-labeled-cell-enriched in the held-out analysis, containing 16 Sensitive-labeled and 86 Resistant-labeled cells (S8 Table and S2 Fig). Despite this imbalance, the model showed strong local score-label separation, with a local AUROC of 0.948 and an AUPRC of 0.703, which was substantially above the local Sensitive-label prevalence baseline of 0.157. Sensitive-labeled cells in this cluster received high predicted Sensitive probabilities, whereas Resistant-labeled cells received low predicted Sensitive probabilities, resulting in a median probability difference of 0.930. The top model-derived pathway deviations were dominated by cell-cycle and mitotic programs, consistent with a cell-cycle-related transcriptional state in this Palbociclib context. Therefore, the high cluster-wise threshold should be interpreted as a post hoc summary of local score-label calibration within a Resistant-labeled-cell-enriched transcriptional neighborhood, rather than as an independently validated biological cutoff. In the Erlotinib case (Fig 4E), cluster-wise optima span both above and below the global cutoff: some clusters favor substantially higher decision thresholds, whereas some lower-threshold neighborhoods lie below the global threshold. This illustrates that a single global decision boundary may summarize cohort-level performance while masking local score-label calibration differences across transcriptional neighborhoods.

The divergence between global and cluster-specific optima highlights a limitation of one-size-fits-all predictors for precision medicine. A fixed cohort-level threshold may achieve strong average metrics yet underperform in identifying clinically relevant resistance-associated neighborhoods concentrated in specific regions [52]. By explicitly mapping subpopulation-aware decision behavior, scRADAR provides a descriptive framework for interpreting response predictions in the presence of ITH and for prioritizing transcriptional neighborhoods for follow-up experimental or clinical annotation. These cluster-wise threshold differences are intended as a post hoc characterization of local decision geometry rather than a threshold adaptation procedure for unlabeled future samples.

### 2.5. Pathway-level attribution highlights candidate signaling and metabolic features associated with Resistant-labeled states

**Fig 5:**
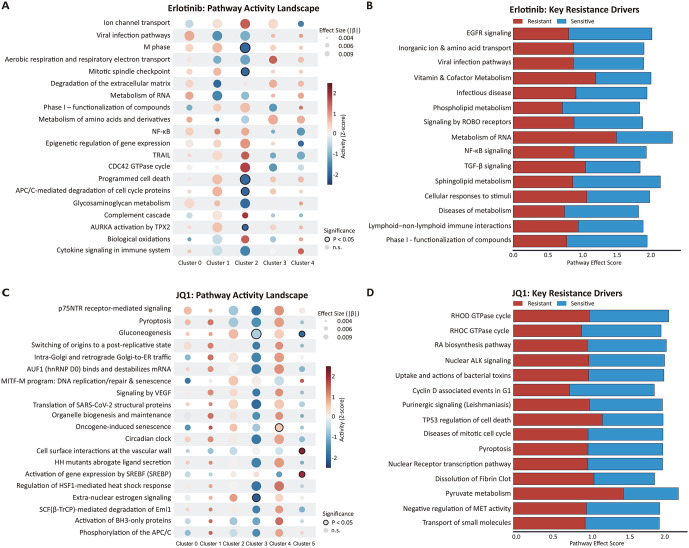
Pathway landscape analysis identifies candidate resistance-associated pathway programs. **(A, C)** Bubble plots showing cluster-wise mean pathway activity (z-scored) for Erlotinib (A) and JQ1 **(C)**, where color indicates pathway activity (Z-score) and circle size denotes effect size (|β|). **(B, D)** Stacked bars summarizing top-ranked pathway effect scores contrasting cells assigned to Resistant (red) versus Sensitive (blue) labels. Note the enrichment of TGF-β-related attribution among Erlotinib-associated Resistant-labeled cells (B) and pyruvate-metabolism-related attribution among JQ1 Resistant-labeled cells **(D)**.

Beyond predictive accuracy, scRADAR provides pathway-level attributions that translate cell-wise response scores into interpretable molecular programs. By aggregating attributions across cells, we derived two complementary insights: cluster-resolved pathway activity landscapes that expose neighborhood-specific programs, as shown in Fig 5A and 5C, and global contrasts between Resistant- and Sensitive-labeled cells that prioritize putative resistance-associated pathways, summarized in Fig 5B and 5D.

In the EGFR-TKI cohort (Erlotinib), the contrast between Resistant- and Sensitive-labeled cells showed elevated TGF-β-related attribution patterns and reduced attribution to canonical EGFR-related signaling features in the Resistant-labeled group (Fig 5B). Importantly, this attribution pattern was accompanied by higher TGF-β-related signals, most notably TGF-β signaling, consistent with previously reported mesenchymal-like and stress-adaptive programs associated with TKI failure, although the present analysis does not establish a causal transition [53,54]. Furthermore, the cluster-level landscape visualized in Fig 5A reveals substantial spatial heterogeneity across neighborhoods. Specific patterns emerge in proliferation-related programs (e.g., M-phase/mitotic checkpoint) and extracellular-matrix–associated processes, suggesting that proliferation-related and microenvironment-associated features vary across the manifold rather than forming a single uniform Resistant-labeled pattern.

Similarly, in the BET-inhibitor cohort (JQ1), cytoskeletal and metabolic signatures emerged as prominent features associated with Resistant-labeled cells. As illustrated in Fig 5D, the top response-associated pathway features characterizing Resistant-labeled cells include Rho-family GTPase cycles (RHOD/RHOC) and pyruvate metabolism, suggesting cytoskeletal plasticity and energetic metabolism as candidate axes for follow-up perturbation-based validation [55]. The cluster-resolved landscape further emphasizes that these programs are not ubiquitously active but rather heterogeneous across neighborhoods. Fig 5C shows that while some clusters exhibit broad attenuation of multiple pathways, others display selective activation of specific modules—such as SREBF-related regulation and vascular interaction signatures—indicating multiple response-associated transcriptional patterns rather than a monolithic Resistant-labeled phenotype.

Collectively, these pathway maps should be interpreted as hypothesis-generating model-derived associations rather than direct experimental evidence of causal mechanisms. They provide a basis for prioritizing candidate response-associated programs for future perturbation-based validation [56]. Accordingly, terms such as “TGF-β-associated” or “metabolic response-associated” are used here to describe retrospective model-derived associations with response-associated labels, not experimentally verified mechanisms of resistance.

### 2.6. Sparse prototype routing exposes archetypal decision modes and preserves interpretability in complex microenvironments

**Fig 6:**
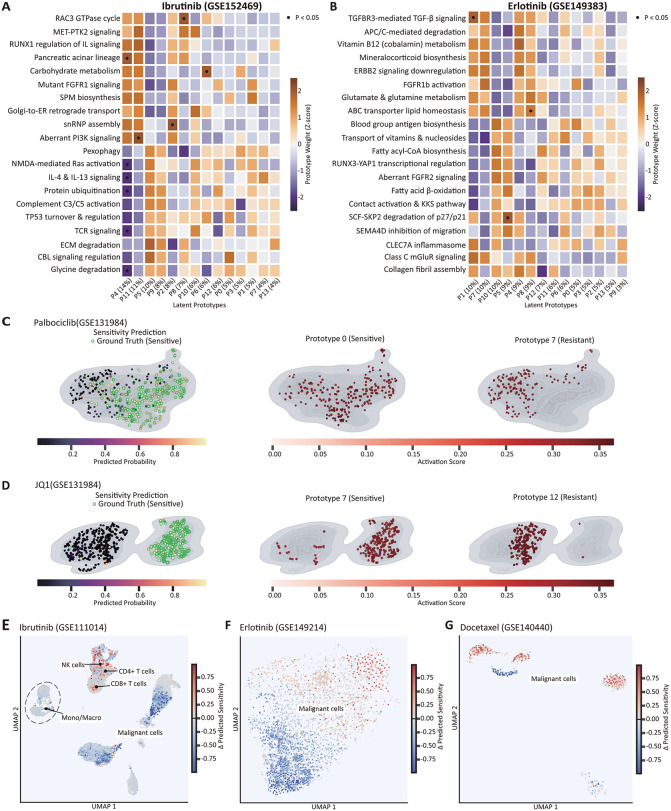
Sparse prototype routing yields interpretable latent response archetypes. **(A,B)** Prototype–pathway weight heatmaps (black dots: P < 0.05). **(C,D)** UMAP maps of per-cell activation for representative prototypes that were post hoc enriched for Sensitive- or Resistant-labeled cells. **(E–G)** Predicted probability of the Sensitive response phenotype projected onto UMAP embeddings across a low-purity microenvironmental sample (E) and malignant-dominant cohorts **(F,G)**, revealing response-probability gradients across transcriptional neighborhoods.

To move beyond the “black-box” perception of deep learning, we dissected the internal decision structure of scRADAR through prototype analysis, as visualized in Fig 6. By examining the learnable connections between latent prototypes and biological pathways, we show that scRADAR organizes high-dimensional transcriptomic variability into a compact set of latent response archetypes that can be characterized by pathway-attribution patterns. The prototype–pathway weight maps provide a concise lookup table linking each latent prototype to characteristic pathway signatures.

In the Erlotinib cohort, Fig 6B highlights that the learned prototypes align with distinct regulatory axes. Specifically, Prototype P1 (~10% of cells) showed elevated attribution to TGFBR3-mediated TGF-β-related features (P<0.05) [54,57]. Beyond this axis, scRADAR identifies separable biological programs: Prototype P8 (~9%) is significantly associated with ABC transporter lipid homeostasis (P<0.05) [58], suggesting a distinct metabolic/efflux-related signature, while Prototype P5 (~9%) shows significant attribution to SCF–SKP2 degradation of p27/p21 (P<0.05), consistent with cell-cycle checkpoint–linked regulation [59]. Turning to the Ibrutinib cohort (Fig 6A), the most prevalent prototype P4 (~14%) shows positive attribution to the pancreatic acinar lineage program (P<0.05) and carries prominent weights on the RAC3 GTPase cycle and MET–PTK2 signaling. Notably, another frequent prototype, P11 (~11%), is associated with aberrant PI3K signaling (P<0.05), indicating a separable PI3K-centered module [60]. Overall, these attribution patterns support the interpretation that the learned prototypes are associated with coherent pathway signatures rather than functioning solely as arbitrary latent routing anchors.

Having established these pathway-defined archetypes, we next projected prototype activations onto the UMAP manifold to examine whether individual prototypes were locally enriched in response-associated transcriptional neighborhoods (Fig 6C and 6D). In the Palbociclib dataset, P0, which was post hoc enriched among Sensitive-labeled cells, showed higher activation in regions with elevated predicted probabilities of the Sensitive response phenotype, whereas P7, which was enriched among Resistant-labeled cells, was activated in regions dominated by Resistant-labeled cells (Fig 6C). In the JQ1 dataset, a similar complementary pattern was observed between P7 and P12: P7 was preferentially activated in regions enriched for Sensitive-labeled cells, whereas P12 was preferentially activated in regions enriched for Resistant-labeled cells (Fig 6D). These observations indicate that the prototype labels are post hoc interpretations based on routing enrichment and associated response labels, rather than predefined biological classes. The resulting topology supports the view that sparse routing organizes cells into multiple latent response archetypes instead of relying on a single global decision rule.

Finally, we further examined whether this structured interpretability remained evident under the confounding complexity of tumor microenvironments. Visualized in Fig 6E–G, scRADAR maintained informative response-probability patterns in low-purity, microenvironment-rich settings. In GSE111014 (Fig 6E), the model preserved heterogeneous gradients in the predicted probability of the Sensitive response phenotype within the malignant compartment while exhibiting coherent, cell-type-resolved patterns across immune and stromal populations. Similar malignant-resolved response-probability gradients were evident in the Erlotinib and Docetaxel cohorts (Fig 6F and 6G). Collectively, these observations suggest that prototype-based routing provides a structured post hoc summary of response-associated pathway patterns in a manner that remains informative under complex microenvironmental admixture.

To further test whether prototype activations corresponded to reproducible response-associated structures rather than arbitrary routing anchors, we quantified response-label and transcriptional-cluster enrichment among the top-routed cells for representative prototypes (S9 Table). Across multiple drug cohorts, selected prototypes showed significant post hoc enrichment for Sensitive- or Resistant-labeled cells and were also enriched within specific transcriptional clusters after FDR correction. For example, Palbociclib P4 was enriched among Resistant-labeled cells and Cluster 0, whereas Palbociclib P6 was enriched among Sensitive-labeled cells and Cluster 2. Similar label- and cluster-enrichment patterns were observed in JQ1, Erlotinib, Ibrutinib, and Cisplatin cohorts. These results support the interpretation that prototypes function as learned latent response archetypes anchored in both pathway-attribution patterns and local transcriptional neighborhoods, while their biological labels remain post hoc summaries rather than predefined classes.

## 3. Discussion

Precision oncology demands models that reconcile predictive accuracy with biological intelligibility. Here, we introduced scRADAR, a mechanism-guided framework that moves single-cell drug-response phenotype modeling beyond black-box classification toward transparent, mechanism-linked reasoning. By integrating dual-view pathway representations with pharmacological priors, scRADAR is more resilient to scRNA-seq sparsity and noise and generalizes consistently across nine independent cohorts. Within our benchmark, it showed strong performance relative to transfer-learning baselines, a scGEN-adapted single-cell perturbation-oriented comparator, and conventional supervised classifiers, particularly on imbalance-sensitive metrics such as AUPRC and F1. The tight performance variation across cross-validation folds further supports robustness to train–test split perturbations, positioning scRADAR as a useful framework for characterizing intratumoral response heterogeneity across single-cell cohorts.

Beyond prediction, scRADAR provides an interpretable lens for examining response-associated heterogeneity. Sparse prototype routing decomposes Resistant-labeled response states into a compact set of post hoc decision modes rather than opaque scalar scores, enabling comparison with canonical pharmacological annotations while retaining flexibility to highlight candidate context-specific pathway signatures. By organizing cells into prototype-defined response-associated modes, the framework supports hypothesis-driven prioritization of subpopulations and pathway programs for future validation.

Several limitations reflect the current state of single-cell pharmacogenomics. Public scRNA-seq response datasets are largely retrospective and heterogeneous in protocols, dosing, and response definitions, and labels often lack a unified functional ground truth, constraining supervised learning. In this study, we addressed this limitation through cohort-specific label harmonization rules summarized in S1 Table; however, some cohorts still rely on operational binary benchmarking labels rather than direct single-cell functional response measurements. The pathway and prototype analyses in this study should therefore be viewed as hypothesis-generating interpretations of retrospective single-cell cohorts rather than direct functional validation of resistance-associated mechanisms. Experimental perturbation or patient-matched functional assays will be required to determine whether the highlighted TGF-β-related, cytoskeletal, or metabolic signatures have causal roles in drug-response phenotypes. The controlled GSE131984 unseen-drug analysis provides a stricter transfer assessment than the within-cohort held-out-cell benchmark by withholding all cells from the test drug during training, validation, threshold selection, and hyperparameter selection. However, this analysis remains within a single study and shared cell-line background. Future work should evaluate cross-study, cross-cancer-type, and prospectively collected perturbation cohorts once sufficiently harmonized labels and comparable experimental designs become available.

Although the pathway-set sensitivity analyses indicated that the main predictive conclusions were stable across Reactome-, Hallmark-, and B-cell-augmented representations, pathway-based models remain constrained by the biological coverage of the selected gene-set libraries. Disease- or lineage-specific signatures may improve interpretive resolution for particular cohorts, but using a common pathway basis remains important for cross-cohort comparability. In addition, curated targets and pathway priors remain incomplete and context dependent, potentially missing non-canonical or microenvironment-driven programs [22,29,38]. This limitation is also reflected in the signaling branch, as the PROGENy view currently relies on only 14 canonical pathways [61], thereby restricting the model’s capacity to capture entirely novel signaling resistance mechanisms outside this predefined pathway space. Finally, drug response is dynamic across time and dose, yet most cohorts provide limited temporal resolution; prospective validation with standardized assays, pre-specified decision thresholds, and patient-matched outcomes remains essential for clinical translation.

Future work will prioritize incorporating spatial transcriptomics to model microenvironmental constraints, extending the dual-view representation to multimodal measurements (*e.g.*, scATAC-seq, surface proteins), and continuously updating the mechanism-aware encoder as perturbation atlases and drug–target networks expand to better support novel agents and combinations.

## 4. Methods

### 4.1. Overview of the scRADAR framework

The scRADAR framework integrates biologically grounded modules into a unified end-to-end pipeline to predict drug-response phenotypes from single-cell transcriptomes (Fig 1). The workflow begins with data acquisition and preprocessing as outlined in Fig 1A, where raw scRNA-seq matrices undergo rigorous quality control and normalization. To mitigate the technical variability inherent in gene-level features, Fig 1B illustrates the construction of a dual-view cellular representation that aggregates metabolic pathway activities—estimated via ssGSEA using GSVA [62] —together with signaling perturbation responses inferred by PROGENy [61]. Parallel to cellular encoding, we synthesize a mechanism-aware drug fingerprint (Fig 1C) by integrating static target annotations with dynamic perturbational signatures. These representations are then coupled via Feature-wise Linear Modulation (FiLM), depicted in Fig 1D, which reweights pathway embeddings in a drug-dependent manner according to the mechanism-aware fingerprint. Finally, as demonstrated in Fig 1E, predictions are generated through interpretable prototype routing, where a query vector maps each cell–drug pair to a sparse set of latent prototypes, decomposing predictions into transparent, biologically meaningful archetypes.

### 4.2. Problem formulation

We formulate single-cell drug-response phenotype prediction as a binary classification problem. Let $𝒟=\overset{N}{\underset{i=1}{\left\{({𝐱}_{i},{𝐯}_{i},y_{i})\right\}}}$ denote a dataset of N single cells. For each cell *i*, ${𝐱}_{i}\in {\mathbb{R}}^{G}$ represents the gene expression profile across *G* genes, and ${𝐯}_{i}$ denotes the mechanism-aware feature vector of the applied drug. The label $y_{i}\in \left\{0,1\right\}$ indicates the cohort-harmonized binary response phenotype with $y_{i}=1$ denoting the Sensitive label and $y_{i}=0$ denoting the Resistant label. Our objective is to learn a parameterized function $f_{\theta }$ that estimates the predicted probability of the Sensitive response phenotype: ${\hat{y}}_{i}=f_{\theta }\left({𝐱}_{i},{𝐯}_{i}\right)\in \left[0,1\right]$. Because the included GEO cohorts use heterogeneous annotation schemes, Sensitive and Resistant denote cohort-harmonized response-associated labels; in longitudinal, treatment-stage, or subset-constructed cohorts, they should be interpreted as operational binary labels for benchmarking rather than direct single-cell functional viability measurements.

### 4.3. Data acquisition and preprocessing

#### 4.3.1. Data acquisition.

Publicly available scRNA-seq drug-response cohorts were collected from the NCBI Gene Expression Omnibus (GEO) repository [63–65]. We curated nine independent cohorts for single-cell drug-response phenotype prediction spanning five cancer types and seven drugs (Table 2), corresponding to GSE111014 [66], GSE117872 [67], GSE149214 [68], GSE149383 [68], GSE152469 [69], GSE140440 [70], and three drug-specific subsets derived from GSE131984 [71]. Cohorts were included based on the following criteria: (1) human cancer-derived single-cell transcriptomes; (2) exposure to a defined small-molecule perturbation; (3) availability of gene-by-cell count matrices with accompanying cell-level annotations; and (4) clearly defined or reproducibly harmonizable binary response-associated labels. Because these GEO cohorts originated from independent studies with heterogeneous annotation schemes, binary response labels were harmonized on a dataset-specific basis using the original study metadata, sample annotations, preprocessing-defined comparison states, and cohort-specific response definitions reported in the corresponding source publications. Study-defined binary groups were used directly when available; otherwise, Sensitive and Resistant denote harmonized operational labels used for binary benchmarking, consistent with prior single-cell drug-response modeling studies that adopted comparable binary response settings for retrospective scRNA-seq cohorts [23,29]. Cohort-level sources of label assignment, supporting references, and detailed harmonization rules are summarized in S1 Table, and the corresponding analysis-ready cohort statistics are reported in Table 2.

**Table 2:** Overview of single-cell drug-response phenotype datasets used in this study. Summary of the scRNA-seq drug-response phenotype cohorts used in this study, including GEO accession, cancer type, drug, and sample source. n_total_ denotes the number of quality-controlled cells retained for analysis, whereas n_sensitive_ and n_resistant_ denote the numbers of cells assigned to the Sensitive and Resistant response-associated labels, respectively, after dataset-specific label harmonization. Cohort-level sources of label assignment and detailed harmonization rules are provided in S1 Table. “Patient-derived” denotes primary clinical samples, whereas “Cell line” denotes in vitro perturbation experiments using the indicated cell line(s). For GSE131984, three drugs were profiled in the same SUM159 model, and counts are reported separately for each drug-specific comparison.

Dataset (GEO)	Cancer type	Drug	Source	n_total_	n_sensitive_	n_resistant_
GSE111014	Chronic lymphocytic leukemia	Ibrutinib	Patient-derived	24790	12145	12645
GSE117872	Oral squamous cell carcinoma	Cisplatin	Patient-derived	1047	695	352
GSE149214	Non-small cell lung carcinoma	Erlotinib	Cell line (PC9)	2386	1108	1278
GSE149383	Non-small cell lung carcinoma	Erlotinib	Cell line (PC9)	2566	1140	1426
GSE152469	Chronic lymphocytic leukemia	Ibrutinib	Patient-derived	9264	4332	4932
GSE140440	Prostate cancer	Docetaxel	Cell lines (DU145, PC3)	284	134	150
GSE131984	Breast cancer	JQ1	Cell line (SUM159)	2603	1057	1546
GSE131984	Breast cancer	Paclitaxel	Cell line (SUM159)	1805	1057	748
GSE131984	Breast cancer	Palbociclib	Cell line (SUM159)	1737	1057	680

#### 4.3.2. Data preprocessing.

We applied a standardized quality control (QC) and normalization pipeline to remove low-quality cells and uninformative genes. For each dataset, mitochondrial genes were identified based on gene symbols, and per-cell QC metrics were computed, including the number of detected genes ($n_{\text{genes}}$), total UMI counts ($n_{UMI}$), and the fraction of mitochondrial transcripts (${\text{pct}}_{\text{mt}}$) [72] within the scverse ecosystem [73]. Cells were retained only if they satisfied $n_{\text{genes}}\geq 200$, $n_{\text{counts}}\geq 200$ and ${\text{pct}}_{\text{mt}}\leq 20\%$. Genes expressed in fewer than 3 cells were removed to reduce noise from extremely sparse features.

Let ${𝐗}^{\text{raw}}$ denote the post-QC raw count matrix, where the entry $\overset{raw}{\underset{ij}{x}}$ represents the raw count of gene *j* in cell *i*. We then performed library-size normalization followed by log-transformation to stabilize variance. Specifically, counts in each cell *i* were normalized to a fixed library size of ${\text{1}\text{0}}^{\text{4}}$ and transformed as


$$\begin{array}{c} x_{i}=log\left(\frac{\overset{raw}{\underset{i}{x}}}{\sum_{j}\overset{raw}{\underset{ij}{x}}}\times \text{1}{\text{0}}^{\text{4}}+\text{1}\right) \end{array}$$ (1)

yielding the normalized expression vector ${𝐱}_{i}$ used for downstream feature construction and model training [74].

Downstream machine-learning utilities for evaluation and model selection were implemented using scikit-learn [75].

### 4.4. Dual-view pathway representation

Given the high sparsity (often exceeding 90% zeros) and high dimensionality of scRNA-seq data, directly using gene-level expression as model input can be unstable and sensitive to technical noise [76]. To obtain a more robust and biologically meaningful description of the cellular state, we construct a dual-view pathway representation that jointly captures metabolic activities and signaling pathway responses for each cell.

#### 4.4.1. Metabolic view (ssGSEA).

For the metabolic view, we quantify pathway activity using single-sample Gene Set Enrichment Analysis (ssGSEA) based on curated gene sets from the Reactome database [77]. Reactome was selected as the default ssGSEA gene-set collection because it provides manually curated and hierarchically organized pathways with broad coverage across cellular processes, which facilitates consistent feature construction and cross-cohort comparison. Nevertheless, a fixed pathway collection cannot capture every disease- or lineage-specific program. Therefore, Reactome-derived ssGSEA scores should be interpreted as a standardized pathway basis rather than an exhaustive representation of all potentially relevant biology. To evaluate whether the main conclusions depended on this pathway choice, we performed pathway-set sensitivity analyses using an alternative Hallmark-based representation, an expanded Reactome + Hallmark representation, and B-cell-specific signatures for GSE111014. Let $S_{m}$ be the gene set corresponding to the *m*-th metabolic pathway. To quantify the activity of this pathway in a specific cell *i*, ssGSEA transforms the cell’s gene expression vector ${𝐱}_{i}$ into a rank-ordered list. It then calculates an enrichment score $ES_{i,m}$ that reflects the relative overrepresentation of genes in $S_{m}$ among highly expressed genes in cell *i* [36,78]. Evaluating ssGSEA across all $N_{\text{meta}}$ selected metabolic pathways yields a metabolic-view embedding:


$$\begin{array}{c} \overset{\text{meta}}{\underset{i}{h}}={\left(ES_{i,\text{1}},\ldots ,ES_{i,N_{\text{meta}}}\right)}^{⊤}\in R^{N_{\text{meta}}} \end{array}$$ (2)

where $N_{\text{meta}}$ denotes the number of metabolic pathways, and $(\cdot {)}^{⊤}$ denotes the transpose operator, so that $\overset{\text{meta}}{\underset{i}{𝐡}}\in {\mathbb{R}}^{N_{\text{meta}}}$ is a column vector.

#### 4.4.2. Signaling view (PROGENy).

To capture the cell’s dynamic potential to respond to drug perturbations, we construct a complementary signaling view using the PROGENy model for pathway activity inference [61].

PROGENy leverages large-scale perturbation transcriptomics to derive a pathway footprint weight matrix $𝐖\in {\mathbb{R}}^{G\times N_{\text{sig}}}$.

Given the gene expression vector ${𝐱}_{i}$, we denote the inferred activity of signaling pathway *p* in cell *i* by $A_{i,p}$ computed as a weighted linear combination:


$$\begin{array}{c} A_{i,p}=\sum_{g=\text{1}}^{G}W_{gp}x_{ig} \end{array}$$ (3)

where $x_{ig}$ denotes the expression of gene *g* in cell *i*, and $W_{gp}$ is the specific weight linkin*g* gene *g* to signaling pathway *p* derived from the PROGENy model. Here, the collection of all weights forms the matrix $𝐖\in {\mathbb{R}}^{G\times N_{\text{sig}}}$, where $N_{sig}$ is the number of pathways. In practice, we use the official human PROGENy wei*g*hts and retain the top 500 genes with the largest absolute weights for each pathway.

Stacking activities across all $N_{sig}$ signaling pathways yields the signaling-view embedding:


$$\begin{array}{c} \overset{\text{sig}}{\underset{i}{h}}={\left(A_{i,\text{1}},\ldots ,A_{i,N_{\text{sig}}}\right)}^{⊤}\in R^{N_{\text{sig}}} \end{array}$$ (4)

#### 4.4.3. Fusion into a unified pathway embedding.

Finally, we fuse the metabolic and signaling views into a unified pathway-level representation for each cell. For each train–validation–test split, pathway-score standardization was fit using only the training cells. The resulting training-set means and standard deviations were then applied unchanged to the corresponding validation and held-out test cells. This fold-specific standardization procedure was used for scRADAR and all baselines requiring scaled features, thereby preventing validation or test-set information from entering feature normalization. We denote this operation by Norm(·), applied independently to $\overset{\text{meta}}{\underset{i}{𝐡}}$ and $\overset{\text{sig}}{\underset{i}{𝐡}}$. The resulting cell-level pathway embedding is obtained by concatenation:


$$\begin{array}{c} \overset{\text{cell}}{\underset{i}{h}}=\text{Concat}\left(Norm(\overset{\text{meta}}{\underset{i}{𝐡}}),Norm(\overset{\text{sig}}{\underset{i}{𝐡}})\right)\in R^{D_{\text{path}}} \end{array}$$ (5)

where $D_{\text{path}}=N_{\text{meta}}+N_{\text{sig}}$ denotes the total dimensionality of the dual-view pathway feature vector. This pathway-level embedding $\overset{\text{cell}}{\underset{i}{𝐡}}$ serves as the input representation for subsequent mechanism-guided modulation and prototype-based prediction in scRADAR. To assess whether the predictive conclusions were sensitive to pathway-feature normalization, we further performed a normalization sensitivity analysis after pathway-score construction. The default setting used training-cell-fitted z-score standardization. Three alternative scaling strategies were evaluated under the same model architecture and data-splitting protocol: robust median/IQR scaling, quantile-normal transformation, and min–max scaling to [0,1]. This analysis was used only to assess preprocessing robustness and was not used for model selection.

### 4.5. Mechanism-aware drug fingerprint

We explicitly encode pharmacological prior knowledge through a mechanism-aware drug fingerprint vector ${𝐯}_{\text{drug}}$. This vector integrates two complementary sources of information: (1) a static fingerprint ${𝐯}_{\text{static}}\in {\mathbb{R}}^{D_{\text{static}}}$, constructed from curated drug–target and pathway annotations provided in the GDSC metadata; and (2) a dynamic fingerprint ${𝐯}_{\text{dynamic}}\in {\mathbb{R}}^{D_{\text{dynamic}}}$, derived from LINCS L1000 small-molecule perturbation signatures and projected into the signaling pathway space via PROGENy. Here, $D_{static}$ and $D_{dynamic}$ denote the dimensionalities of the static and dynamic fingerprints, respectively.

To improve reproducibility, drug identifiers were harmonized before constructing these fingerprints. Drug names from the single-cell cohorts were standardized by converting them to lowercase, unifying spacing and punctuation variants, and removing non-informative salt-form descriptors or vendor-specific suffixes only when the underlying active compound identity was unchanged. The standardized names were then matched to curated GDSC drug annotations to obtain ${𝐯}_{\text{static}}$. For the dynamic component, the same standardized names were matched to LINCS L1000 perturbagen names and aliases. When a direct name match was not available, aliases were manually checked, and only unambiguous matches referring to the same active compound were retained. When multiple eligible LINCS signatures were available for the same compound, small-molecule perturbation signatures with mappable gene identifiers were summarized by an unweighted mean at the compound level before projection into the PROGENy signaling pathway space to obtain ${𝐯}_{\text{dynamic}}$. Before fusion, ${𝐯}_{\text{static}}$ and ${𝐯}_{\text{dynamic}}$ were independently normalized so that their scales were comparable. When one component was unavailable after matching, the available component was retained, and the missing component was represented by a zero vector in the normalized feature space so that all drugs shared the same final fingerprint dimensionality. To construct the final hybrid representation, we employ a weighted concatenation strategy:


$$\begin{array}{c} v_{\text{drug}}=Concat(\lambda_{static}\cdot v_{static},\lambda_{dynamic}\cdot v_{dynamic})\in R^{D_{\text{static}}+D_{\text{dynamic}}} \end{array}$$ (6)

where $\lambda_{\text{static}}$ and $\lambda_{\text{dynamic}}$ are scalar hyperparameters controlling the relative scaling of static and dynamic features, respectively, and Concat(⋅) denotes vector concatenation. Unless otherwise specified, we set $\lambda_{\text{static}}=\lambda_{\text{dynamic}}=1$ in our experiments, thereby preserving the full dimensionality of both information sources while allowing for differential re-weighting if needed.

### 4.6. Feature-wise linear modulation (FiLM)

To model the conditional influence of drug mechanisms on cellular state, scRADAR incorporates a Feature-wise Linear Modulation (FiLM) layer [35]. Given the hybrid drug fingerprint ${𝐯}_{i}$ for the drug applied to cell *i*, a multilayer perceptron ${\text{MLP}}_{\text{FiLM}}$ generates channel-wise affine parameters:


$$\left[\gamma_{i},\beta_{i}]=\text{ML}{\text{P}}_{\text{FiLM}}(v_{i}\right)$$ (7)

where $\gamma_{i}$ and $\beta_{i}$ correspond to the scaling and bias coefficients specific to sample *i*, respectively. In other words, the MLP maps the drug-fingerprint space to the fixed dimensionality of the cellular pathway embedding.

These parameters dynamically recalibrate the cellular embedding $\overset{\text{cell}}{\underset{i}{𝐡}}$ via an affine transformation:


$$\begin{array}{c} \overset{\text{cond}}{\underset{i}{z}}=\gamma_{i}⊙\overset{cell}{\underset{i}{h}}+\beta_{i} \end{array}$$ (8)

where ⊙ denotes element-wise multiplication. Intuitively, FiLM acts as a drug-dependent pathway reweighting step. The cellular pathway embedding first describes which metabolic and signaling programs are active in a given cell. The drug fingerprint then generates scaling and shifting coefficients that increase or decrease the contribution of these pathway features according to the drug’s curated targets and perturbation-derived signatures. Therefore, the same cellular pathway state can be interpreted differently under different drug mechanisms before being routed to the prototype layer. In this study, FiLM is used as a mechanism-level conditioning layer rather than as a fully cell-adaptive interaction module. This design captures drug-dependent reweighting of cell-state features, but it does not model all higher-order context-specific drug–cell interactions. The conditioned representation $\overset{\text{cond}}{\underset{i}{𝐳}}$ is then passed to the prototype routing module for phenotype prediction.

### 4.7. Interpretable prototype routing

To decompose black-box predictions into a set of interpretable patterns, scRADAR employs a sparse prototype routing layer. We introduce a learnable prototype bank:


$$\begin{array}{c} P=\{p_{j}\overset{L}{\underset{j=\text{1}}{\}}}\subset R^{D_{\text{path}}} \end{array}$$ (9)

where 𝒫 consists of L latent prototype vectors, and ${𝐩}_{j}$ denotes the *j*-th prototype representing a distinct molecular pattern. $D_{\text{path}}$ is the dimensionality of the FiLM-conditioned cell–drug embedding.

#### 4.7.1. Top-*k* sparse routing.

For each cell–drug pair, the FiLM-conditioned pathway representation $\overset{\text{cond}}{\underset{i}{𝐳}}\in {\mathbb{R}}^{D_{\text{path}}}$ is first mapped to a query vector:


$$\begin{array}{c} q_{i}=W_{q}\overset{cond}{\underset{i}{z}}+b_{q},\;W_{q}\in R^{D_{path}\times D_{path}},\;b_{q}\in R^{D_{\text{path}}}\; \end{array}$$ (10)

where ${𝐪}_{i}$ denotes the query vector for sample *i* (representing the conditioned state of the cell), and ${𝐖}_{q}$ and ${𝐛}_{q}$ are the learnable projection matrix and bias, respectively.

We then compute the similarity between the query ${𝐪}_{i}$ and each prototype ${𝐩}_{j}$. To ensure that routing is based on pattern alignment rather than magnitude, we employ cosine similarity:


$$\begin{array}{c} s_{ij}=\frac{\overset{⊤}{\underset{i}{q}}p_{j}}{\|q_{i}{\|}_{\text{2}}\|p_{j}{\|}_{\text{2}}} \end{array}$$ (11)

where $s_{ij}$ denotes the cosine similarity between cell *i*’ s query and prototype *j* and $∥\cdot {∥}_{\text{2}}$ denotes the *L*_2_-norm. To encourage sparse and decisive routing, we retain only the top-k most similar prototypes for each cell and denote their index set by ${𝒦}_{i}\subseteq \{1,\ldots ,L\}$, where k∈ℕ is a sparsity hyperparameter and *L* is the total number of prototypes. Routing weights $\alpha_{ij}$ are then obtained by applying a temperature-scaled softmax restricted to ${𝒦}_{i}$:


$$\begin{array}{c} \alpha_{ij}=\left\{ \begin{array}{cc} \frac{\exp(s_{ij}/\tau )}{\sum_{l\in {𝒦}_{i}}\exp(s_{il}/\tau )}, & j\in {𝒦}_{i},\;\tau >0,\\ 0, & otherwise. \end{array}\right. \end{array}$$ (12)

Here, τ is a temperature hyperparameter controlling the sharpness of the routing distribution, and $l\in {𝒦}_{i}$ indexes prototypes within the selected set. By construction, the routing weights satisfy $\sum_{j\in K_{i}}\alpha_{ij}=\text{1}$ [79].

#### 4.7.2. Prototype-based prediction head.

Each prototype ${𝐩}_{j}$ is associated with a learnable scalar response logit $r_{j}\in \mathbb{R}$. The final predicted response probability ${\hat{y}}_{i}$ for cell *i* is then computed as a weighted sum of prototype-specific logits followed by a sigmoid:


$$\begin{array}{c} {\hat{y}}_{i}=\sigma \left(\sum_{j=\text{1}}^{L}\alpha_{ij}r_{j}\right) \end{array}$$ (13)

where σ(·) denotes the sigmoid function. This design ensures that each prediction can be traced back to a small subset of highly activated prototypes, providing an interpretable decomposition of the model’s decision in terms of latent response archetypes.

### 4.8. Loss function and optimization

We train scRADAR end-to-end by minimizing a composite objective that balances predictive accuracy and prototype diversity:


$$\begin{array}{c} L_{total}=L_{BCE}+\lambda_{div}L_{div} \end{array}$$ (14)

where ${ℒ}_{BCE}$ denotes the prediction loss, ${ℒ}_{div}$ represents the prototype diversity regularization, and $\lambda_{div}>0$ is a scalar hyperparameter controlling the trade-off between the two terms.

Model parameters are optimized using the Adam optimizer [80]. Key optimization settings, such as the learning rate and batch size, are treated as tunable hyperparameters.

The prototypes are not predefined Sensitive or Resistant classes. They are randomly initialized learnable latent anchors optimized jointly with the prediction loss and the prototype-diversity regularization. Their biological interpretation is assigned only after training. Specifically, a prototype can be described as Sensitive-enriched or Resistant-enriched only when cells with high routing weights to that prototype show post hoc enrichment of the corresponding cohort-harmonized response-associated label. Thus, prototype labels should be interpreted as summaries of learned latent response archetypes rather than as predefined biological categories.

#### 4.8.1. Sample-weighted binary cross-entropy.

Let $o_{i}$ denote the logit output of the prototype-based prediction head for cell *i*, and let $y_{i}\in \left\{0,1\right\}$ be the corresponding ground-truth response-associated label. The per-sample binary cross-entropy loss is:


$$\begin{array}{c} l_{i}=-\left[y_{i}\;log\;\sigma (o_{i})+(\text{1}-y_{i})\;log\;(\text{1}-\sigma (o_{i}))\right] \end{array}$$ (15)

where σ(·) is the sigmoid function. To handle label imbalance and incorporate sample-level importance, we apply a non-negative weight $\omega_{i}$ to each sample (provided as batch_weights in the implementation) and minimize the weighted average loss [81]:


$$\begin{array}{c} L_{BCE}=\frac{\text{1}}{N}\sum_{i=\text{1}}^{N}\omega_{i}l_{i} \end{array}$$ (16)

In practice, ${ℒ}_{BCE}$ is implemented using a numerically stable binary cross-entropy function with reduction disabling, allowing for element-wise weighting by sample weights $\omega_{i}$.

#### 4.8.2. Prototype diversity regularization.

To prevent mode collapse and encourage a diverse prototype bank, we penalize the squared cosine similarity between different prototypes. Let $𝐏\in {\mathbb{R}}^{L\times D_{\text{latent}}}$ denote the matrix whose rows are prototype vectors ${𝐩}_{j}$. We first apply row-wise *L*_2_-norm to obtain $\tilde{𝐏}$, and form the Gram matrix $𝐆=\tilde{𝐏}{\tilde{𝐏}}^{⊤}$, where ${𝒢}_{jl}$ equals the cosine similarity between prototypes ${𝐩}_{j}$ and ${𝐩}_{l}$. The diversity loss is defined as:


$$\begin{array}{c} {ℒ}_{div}=\frac{\text{2}}{L(L-1)}\sum_{1\leq j<l\leq L}\overset{\text{2}}{\underset{jl}{𝒢}}=\frac{\text{2}}{L(L-1)}\sum_{1\leq j<l\leq L}{cos}^{\text{2}}\left(p_{j},p_{l}\right) \end{array}$$ (17)

which corresponds to the mean squared cosine similarity across all pairs of distinct prototypes. Minimizing ${ℒ}_{div}$ discourages highly correlated prototypes and thus promotes a more expressive and non-redundant prototype set.

### 4.9. Experimental design and evaluation protocol

#### 4.9.1. Group-aware data partitioning.

To rigorously assess generalization and avoid data leakage, we adopt a two-stage, group-aware data partitioning strategy. For each dataset, we first perform an external split into a training set and an independent test set using group-aware shuffling. Whenever metadata provide biological grouping variables (e.g., patient IDs, donor, sample, or batch identifiers), we treat each group as an indivisible unit and perform random partitioning at the group level to assign approximately 80% of the groups to the training split and 20% to the held-out test split. This guarantees that cells originating from the same biological source never appear simultaneously in training and test sets. In datasets where such grouping variables are absent or where the number of groups is too small to support a meaningful group-based split, we fall back to stratified random sampling, which preserves separation between Sensitive- and Resistant-labeled cells while still maintaining a disjoint test set.

#### 4.9.2. Cross-validation and robustness assessment.

All hyperparameter tuning and model selection were conducted exclusively within the training split to prevent data leakage. We employed a group-aware *K*-fold cross-validation strategy (default *K* = 5) on the training partition, ensuring that all cells originating from the same patient or biological sample were confined to a single fold. In cases where group numbers were insufficient for five folds, we adaptively reduced *K* while preserving group integrity; if no grouping variable was available, we utilized stratified *K*-fold cross-validation to maintain class balance.

To quantify model robustness against training data variations, we retained the models generated from the *K* folds (with hyperparameters fixed) and evaluated each model independently on the fixed held-out test set. Performance metrics are reported as the mean and 95% t-interval derived from these *K* inferences, reflecting training-induced variability (e.g., from data splitting and initialization). This interval $I_{0.95}$ is calculated as:


$$\begin{array}{c} I_{\text{0}.\text{9}\text{5}}=\bar{x}\pm t_{\text{0}.\text{9}\text{7}\text{5},\;K-\text{1}}\frac{s}{\sqrt{K}} \end{array}$$ (18)

where *s* is the standard deviation of the metric across the *K* runs/folds and *K* is the number of folds.

#### 4.9.3. Threshold selection.

As described in the loss function section, scRADAR is trained to minimize a weighted composite objective. After training, the raw logit outputs are converted to probabilities via the sigmoid function. To handle potential class imbalance, we optimize the decision threshold adaptively for each cross-validation fold rather than using a default of 0.5.

Specifically, for each fold *f*, we perform a one-dimensional grid search over the candidate thresholds δ∈{0.00,0.05,…,0.95} and select the threshold $\overset{*}{\underset{f}{\delta }}$ that maximizes the F1-score on the corresponding validation subset:


$$\begin{array}{c} \overset{*}{\underset{f}{\delta }}=\;argmax{\;}_{\delta \in [\text{0},\text{1}]}\text{F1}\left(\overset{\left(f\right)}{\underset{\text{val}}{y}},1\left(\overset{\left(f\right)}{\underset{\text{val}}{\hat{y}}}>\delta \right)\right) \end{array}$$ (19)

where $\overset{\left(f\right)}{\underset{\text{val}}{y}}$ denotes the ground-truth labels and $\overset{\left(f\right)}{\underset{\text{val}}{\hat{y}}}$ denotes the predicted probabilities for the *f*-th validation fold, and 1(·) is the indicator function. The optimal threshold $\overset{*}{\underset{f}{\delta }}$ is then applied to the predictions of the *f*-th model on the held-out test set. For prospective inference on unlabeled future samples, however, the decision threshold should be pre-specified from development data rather than re-optimized on the incoming sample. In practice, a stable choice is to use the median of the fold-wise optimal thresholds estimated during model development.

#### 4.9.4. Additional controlled within-study unseen-drug transfer analysis.

In addition to the primary within-cohort held-out-cell evaluation, we performed a controlled within-study unseen-drug analysis using the three drug-specific subsets of GSE131984. This analysis was designed to assess whether the model could transfer across transcriptionally distinct drug responses while reducing confounding from study, platform, and cell-line differences. In each split, two drug-specific subsets were used for training and the remaining drug was held out for testing. No cells from the held-out drug were used for model training, validation, threshold selection, or hyperparameter selection.

To separate the contribution of drug information and drug-cell interaction modeling, four models were compared under the same cellular pathway feature space and threshold-selection protocol. The cell-only MLP used only the cellular pathway representation. The concatenation MLP used the cellular pathway representation concatenated with the mechanism-aware drug fingerprint. The Hadamard-interaction MLP used an element-wise drug-cell interaction term as a generic nonlinear interaction baseline. Full scRADAR used FiLM-conditioned pathway embeddings followed by sparse prototype routing. For all models, thresholds were selected only on validation cells from the training drugs. Results were reported as mean values with 95% t-intervals across five random validation splits.

### 4.10. Baseline implementation and benchmarking protocol

To provide a rigorous and comparable benchmark, we evaluated scRADAR against three categories of comparison methods within a unified target-domain evaluation framework. The first category comprised five representative bulk-to-single transfer-learning or domain-adaptation baselines, namely SCAD [30], scATD [31], scDEAL [23], SSDA4Drug [29], and scAdaDrug [24]. Following their original formulations, these methods used bulk RNA-seq data as the source domain and scRNA-seq cohorts as the target domain, with input features aligned by the intersection of shared genes. Source-domain composition followed each method’s official setting: SCAD, SSDA4Drug, and scAdaDrug were trained using GDSC bulk RNA-seq only, whereas scDEAL and scATD additionally incorporated CCLE bulk profiles (i.e., a GDSC+CCLE integrated source), as supported by their original implementations.

The second category was a scGEN-adapted baseline implemented as a single-cell perturbation-oriented comparator. Because the curated cohorts in this study provide cohort-harmonized binary response-associated labels rather than paired perturbation trajectories for every drug condition, scGEN was adapted as a scGEN-style variational latent representation learner using the same cellular pathway representation. The learned latent representation was then used for downstream binary response-phenotype classification under the same target-domain data-splitting and threshold-selection protocol. Mechanism-aware drug fingerprints were not added to this baseline because they are not native to the scGEN-style latent representation setting.

The third category comprised conventional supervised classifiers trained directly on the target single-cell data, including logistic regression (LR) [44], multilayer perceptron (MLP) [46], and XGBoost [47]. These models were trained using the same target-domain training data as scRADAR, without any bulk pretraining, domain adaptation, or external source-domain supervision.

For each target cohort, we first created a group-aware 80/20 holdout split, strictly separating patients, donors, samples, or batches whenever such metadata were available, to define a fixed independent test set. We then performed 5-fold group-aware cross-validation on the remaining 80% training portion for hyperparameter tuning and model selection. In datasets with insufficient numbers of biological groups, we reduced the number of folds while preserving group integrity; when no grouping variable was available, we used stratified K-fold splitting.

To ensure comparability, all methods—transfer-learning baselines, the scGEN-adapted baseline, supervised baselines, and scRADAR—were evaluated under the same target-domain splits, preprocessing pipeline, and decision-threshold protocol. Feature standardization (z-score normalization), when required by a given model, was fit strictly on the training data available within each fold and then applied to the corresponding validation fold and held-out test set, thereby preventing information leakage. For transfer-learning methods, preprocessing was performed using the source-domain data together with the target training fold in a manner consistent with their original design; for target-only supervised models and scRADAR, preprocessing was fit using the target training fold only. Predicted probabilities were converted to binary labels using the same adaptive thresholding procedure for all methods: the decision threshold was selected by grid search to maximize validation F1-score within each fold and then fixed for evaluation on the held-out test set. Final performance is reported as the mean and 95% t-interval across cross-validation-derived runs.

This benchmark design helps disentangle improvements associated with target-domain supervision, single-cell perturbation-style latent representation learning, and scRADAR’s mechanism-guided architecture and prototype-routing design.

### 4.11. Post hoc interpretability analyses

#### 4.11.1. Model-derived drug-fingerprint score analysis.

After model training, we performed a post hoc analysis of drug-fingerprint score profiles to compare static prior-derived mechanism scores with dynamic perturbation-derived pathway scores for representative drug cohorts. This analysis was used only for interpretation and visualization and was not used for model training, model selection, threshold optimization, or hyperparameter tuning.

For each representative drug cohort, the exported drug-mechanism profile contained two signed score sources. The static score for mechanism or pathway feature *m* is denoted by $\overset{\text{static}}{\underset{m}{u}}$, and the dynamic score for the same feature is denoted by $\overset{\text{dynamic}}{\underset{m}{u}}$. Static scores were derived from curated mechanism annotations, whereas dynamic scores were derived from perturbation-associated pathway profiles represented in the model diagnostic output. Static and dynamic entries referring to the same mechanism or pathway label were mapped to a shared display label. Entries labelled as “Other” or uninformative miscellaneous categories were excluded from visualization. When a feature was present in only one source, its score in the other source was set to zero for visualization.

To focus the plot on the most informative mechanism or pathway features, features were ranked by the larger absolute magnitude of the two score sources:


$$\begin{array}{c} s_{m}=max\left(\left|\overset{\text{static}}{\underset{m}{u}}\right|,\left|\overset{\text{dynamic}}{\underset{m}{u}}\right|\right) \end{array}$$ (20)

The top-ranked features were retained as the displayed feature set for drug cohort *d*, denoted by ${ℳ}_{d}$. Because static and dynamic scores originate from different information streams and may have different numerical scales, they were standardized separately within each drug-specific displayed profile. For the static source, we computed


$$\begin{array}{c} \overset{\text{static}}{\underset{m}{\tilde{u}}}=\frac{\overset{\text{static}}{\underset{m}{u}}-\overset{\text{static}}{\underset{d}{\mu }}}{\overset{\text{static}}{\underset{d}{\sigma }}+\epsilon },\;m\in M_{d} \end{array}$$ (21)

where $\overset{\text{static}}{\underset{d}{\mu }}$ and $\overset{\text{static}}{\underset{d}{\sigma }}$ are the mean and standard deviation of the static scores across ${ℳ}_{d}$, and ε is a small constant for numerical stability. The dynamic scores were standardized analogously:


$$\begin{array}{c} \overset{dynamic}{\underset{m}{\tilde{u}}}=\frac{\overset{dynamic}{\underset{m}{u}}-\overset{dynamic}{\underset{d}{\mu }}}{\overset{dynamic}{\underset{d}{\sigma }}+\epsilon },\;m\in M_{d} \end{array}$$ (22)

The paired values $\overset{\text{static}}{\underset{m}{\tilde{u}}}$ and $\overset{\text{dynamic}}{\underset{m}{\tilde{u}}}$ were then visualized in Fig 3 as static prior-derived and dynamic perturbation-derived drug-fingerprint scores, respectively.

These scores should be interpreted as post hoc diagnostic summaries of the hybrid drug-fingerprint module rather than as independent experimental measurements of pathway activation or causal feature attributions. Static scores summarize curated prior mechanism information, whereas dynamic scores summarize perturbation-derived pathway information represented in the model diagnostic output. Therefore, deviations between static and dynamic scores, such as the Hormone Ligand Binding signal in the Cisplatin cohort, were interpreted as candidate context-specific associations only when they were biologically plausible and supported by external literature. Conversely, negative or discordant scores, including RTK signaling or DNA-crosslinker-related features, were treated as directional differences within the diagnostic profile and interpreted cautiously rather than as direct evidence of pathway inhibition or activation.

#### 4.11.2. Post hoc cluster-wise threshold analysis.

Cluster-wise threshold analysis was performed only as a post hoc characterization of local score-label relationships across transcriptional neighborhoods. It was not used for model training, model selection, global threshold selection, or prospective prediction. The model first produced a continuous predicted probability ${\hat{y}}_{i}$ of the Sensitive response phenotype for each cell *i*. A global decision threshold was selected on the validation set according to the procedure described in Section 4.9.3 and was then fixed for held-out test evaluation.

To examine whether different transcriptional neighborhoods exhibited different local calibration patterns, cells were assigned to the transcriptional clusters visualized in Fig 4. Let *c* denote a cluster and let ${ℐ}_{c}$ denote the set of cells assigned to that cluster. For each cluster, we searched the same candidate-threshold grid used in the global thresholding procedure: 𝒯={0.00,0.05,…,0.95}.

The post hoc cluster-wise threshold was defined as a threshold that maximized the F1-score within that cluster:


$$\begin{array}{c} \overset{*}{\underset{c}{t}}\in \underset{t\in T}{\text{arg}\max}\;F_{1}\left(\{y_{i}{\}}_{i\in I_{c}},\{1({\hat{y}}_{i}\geq t){\}}_{i\in I_{c}}\right) \end{array}$$ (23)

where $y_{i}\in \left\{0,1\right\}$ denotes the cohort-harmonized response-associated label, with $y_{i}=1$ corresponding to the Sensitive label and $y_{i}=0$ corresponding to the Resistant label, ${\hat{y}}_{i}$ denotes the predicted probability of the Sensitive response phenotype, and 1(·) is the indicator function. Clusters with small cell numbers were interpreted cautiously because their F1-optimizing thresholds can be unstable.

Because $\overset{*}{\underset{c}{t}}$ was estimated using cluster-specific labels after model evaluation, it should not be interpreted as a deployable decision rule for unlabeled future samples. Instead, it summarizes how the relationship between predicted probabilities and response-associated labels varies across transcriptional neighborhoods. A cluster-wise threshold higher than the global threshold indicates that cells in that neighborhood require a higher predicted Sensitive probability to achieve the best local F1-score, whereas a lower threshold indicates a different local score-label calibration pattern. These thresholds were therefore used to visualize heterogeneity in local decision geometry, not to claim independently validated biological subtypes or to adapt predictions in a prospective setting.

#### 4.11.3. Cluster-level response-label enrichment analysis.

To statistically support the descriptive response-landscape analysis in Fig 4A–C, we performed cluster-level response-label enrichment tests. For each transcriptional cluster, the observed Sensitive-label fraction was compared with a permutation-derived null distribution generated by randomly permuting response-associated labels while preserving cluster size and cohort-level label composition. Empirical P values were estimated using 5,000 label permutations and adjusted using the Benjamini–Hochberg procedure within each cohort. Clusters were annotated as Sensitive-enriched or Resistant-enriched when the observed Sensitive-label fraction was above or below the cohort-level Sensitive-label fraction, respectively, and the permutation FDR q-value was below 0.05. This analysis was used only to support post hoc interpretation of transcriptional neighborhoods and was not used for model training, threshold selection, or prediction.

## Supporting information

S1 Fig Cross-view correlation analysis supports the complementarity of metabolic and signaling pathway representations. (A) Cohort-level summary of cross-view Pearson correlations between ssGSEA-derived metabolic features and PROGENy-derived signaling features across the nine analyzed GEO cohorts. Blue circles denote the overall mean absolute correlation (mean |r|), orange squares denote the overall median absolute correlation (median |r|), and green diamonds denote the median feature-wise best-match absolute correlation. (B) Representative cross-view correlation matrix for GSE152469, showing pairwise Pearson correlations between the displayed metabolic pathways and the 14 PROGENy signaling pathways. (C) Top metabolic-to-signaling pathway associations in GSE152469, ranked by best-match absolute correlation (|r|). (D) Distribution of absolute cross-view Pearson correlations in GSE152469. Vertical lines indicate the overall mean |r|, overall median |r|, and median best-match |r|. Collectively, these analyses indicate modest overall cross-view correlation together with a limited subset of stronger local associations, supporting the view that the two pathway representations are complementary rather than broadly redundant.
(EPS)

S2 Fig Post hoc characterization of Palbociclib Cluster 0. (A) UMAP visualization highlighting Cluster 0 in the Palbociclib cohort. (B) Predicted Sensitive probabilities of Resistant-labeled and Sensitive-labeled cells within Cluster 0, showing clear local score-label separation. AUROC, AUPRC, and the Mann–Whitney test P value were computed using held-out cells in this cluster. (C) Top model-derived Reactome pathway deviations in Cluster 0, dominated by cell-cycle and mitotic programs. These analyses were performed only for post hoc interpretation and were not used for model training, threshold selection, or prospective prediction.
(TIF)

S1 Table Cohort-level source of label assignment and dataset-specific harmonization rules used to derive Sensitive versus Resistant labels across the nine GEO cohorts. For cohorts with explicit study-defined binary response groups, those labels were used directly. For longitudinal, treatment-stage, or subset-constructed cohorts, Sensitive and Resistant denote harmonized operational labels used for binary benchmarking rather than direct single-cell functional viability measurements.
(DOCX)

S2 Table Normalization sensitivity analysis of full scRADAR. Full scRADAR was evaluated under four pathway-feature normalization settings using the same model architecture, data-splitting protocol, and threshold-selection procedure. The default setting corresponds to training-cell-fitted z-score standardization, in which scaling parameters were estimated only from the training cells and then applied unchanged to validation and held-out test cells. The other settings were evaluated only for sensitivity analysis and were not used for model selection. Values are shown as mean ± 95% t-interval across cross-validation-derived runs.
(DOCX)

S3 Table Pathway-view and pathway-set sensitivity analysis of scRADAR. The default full scRADAR setting used Reactome ssGSEA and PROGENy as the dual-view pathway representation. For sensitivity analysis, the pathway representation was modified while the drug fingerprint, FiLM conditioning, prototype-routing module, data-splitting protocol, and threshold-selection procedure were kept unchanged. The ssGSEA-only and PROGENy-only variants removed one pathway view to assess the contribution of each view separately. The Hallmark and Reactome + Hallmark settings were evaluated to test whether performance depended on the selected ssGSEA gene-set collection. These sensitivity analyses were not used for model selection. Values are shown as mean ± 95% t-interval across cross-validation-derived runs.
(DOCX)

S4 Table GSE111014 B-cell pathway-signature sensitivity analysis. B-cell-specific signatures were evaluated only in the GSE111014 Ibrutinib cohort as a disease-context sensitivity analysis. The default setting used Reactome ssGSEA and PROGENy, whereas the augmented settings added B-cell-specific ssGSEA signatures, with or without Hallmark ssGSEA features. B-cell-specific signatures included CD40 signaling, NF-κB signaling, B-cell receptor signaling, BTK downstream signaling, B-cell activation, apoptosis, antigen presentation, and proliferation-related programs. The full scRADAR architecture, drug fingerprint, FiLM conditioning, prototype-routing module, data-splitting protocol, and threshold-selection procedure were kept unchanged across all settings. Retained total features indicate the final number of pathway-level input features after pathway scoring and feature construction; retained B-cell signatures indicate the number of B-cell-specific signatures included in the feature set. These sensitivity analyses were not used for model selection. Values are shown as mean ± 95% t-interval across cross-validation-derived runs.
(DOCX)

S5 Table scGEN-adapted baseline comparison. The scGEN-adapted baseline was included as a representative single-cell perturbation-oriented comparator. Because the curated cohorts provide cohort-harmonized binary response-associated labels rather than paired perturbation trajectories for every drug condition, scGEN was adapted as a scGEN-style variational latent representation learner using the same Reactome ssGSEA + PROGENy cellular pathway representation as the base input. The learned latent representation was then used for downstream binary response-phenotype classification under the same target-domain data-splitting and threshold-selection protocol. XGBoost is shown as a strong target-domain supervised comparator, whereas scRADAR represents the full proposed model. Values are shown as mean ± 95% t-interval across cross-validation-derived runs.
(DOCX)

S6 Table Controlled within-study unseen-drug transfer and drug-cell interaction ablation in GSE131984. In each split, two GSE131984 drug-specific subsets were used for training and the remaining drug was held out for testing. No cells from the held-out drug were used for model training, validation, threshold selection, or hyperparameter selection. Cell-only MLP used the shared cellular pathway representation. Concat MLP used the cellular pathway representation concatenated with the mechanism-aware drug fingerprint. Hadamard MLP used an element-wise drug-cell interaction term to evaluate a generic nonlinear drug-cell interaction baseline. Full scRADAR used FiLM-conditioned pathway embeddings followed by sparse prototype routing. Sensitive-label prevalence indicates the fraction of Sensitive-labeled cells in the held-out drug subset. Values are shown as mean ± 95% t-interval across five random validation splits. Macro averages were computed across the three held-out drugs using unrounded held-out-drug means.
(DOCX)

S7 Table Cluster-level response-label enrichment analysis for Fig 4A–C. Cluster-level enrichment was evaluated using the cells included in the post hoc Fig 4A–4C analysis by comparing the observed Sensitive-label fraction in each transcriptional cluster with a permutation-derived null distribution that preserved cluster size and cohort-level label composition. Empirical P values were estimated using 5,000 label permutations and adjusted using the Benjamini–Hochberg procedure within each cohort. Clusters were annotated as Sensitive-enriched or Resistant-enriched when the observed Sensitive-label fraction was above or below the cohort-level Sensitive-label fraction, respectively, and the permutation FDR q-value was below 0.05. Clusters with fewer than 20 cells were interpreted cautiously because enrichment estimates are more sensitive to sampling variation.
(DOCX)

S8 Table Post hoc characterization of Palbociclib Cluster 0. Palbociclib Cluster 0 was characterized using held-out cells assigned to this transcriptional neighborhood. AUROC and AUPRC were computed from predicted Sensitive probabilities and cohort-harmonized response-associated labels. Because Cluster 0 was Resistant-labeled-cell-enriched and contained only 16 Sensitive-labeled cells, AUPRC was interpreted relative to the local Sensitive-label prevalence baseline. The Mann–Whitney test compared predicted Sensitive probabilities between Sensitive-labeled and Resistant-labeled cells within this cluster. Reactome pathway deviations were computed from model-derived pathway activity profiles. These analyses were used only for post hoc characterization and were not used for model training, threshold selection, or prospective prediction.
(DOCX)

S9 Table Representative post hoc characterization of learned prototypes. Representative prototypes were characterized after model training using cells with the highest routing weights to each prototype. Response-label enrichment was evaluated by testing whether the top-routed cells were enriched for Sensitive-labeled or Resistant-labeled cells relative to the corresponding cohort background. Transcriptional-cluster enrichment was evaluated by testing whether the same top-routed cells were overrepresented in specific transcriptional clusters. FDR q-values were obtained after multiple-testing correction. Prototype labels such as Sensitive-labeled enriched or Resistant-labeled enriched were assigned only as post hoc summaries of enriched routed cells; the prototypes were not predefined as Sensitive or Resistant classes during model training. These analyses were used only for interpretability assessment and were not used for model training, model selection, threshold selection, or prospective prediction.
(DOCX)
