# Supplementary material for: scRADAR: Dissecting intratumoral drug response heterogeneity at single-cell resolution via mechanism-guided prototype routing
Source: PLoS Comput Biol. 2026 Jun 26;22(6):e1014392. doi: 10.1371/journal.pcbi.1014392 (PMC13309031; doi:10.1371/journal.pcbi.1014392)
Supplement: S1 Table — For cohorts with explicit study-defined binary response groups, those labels were used directly. For longitudinal, treatment-stage, or subset-constructed cohorts, Sensitive and Resistant denote harmonized operational labels used for binary benchmarking rather than direct single-cell functional viability measurements. (DOCX) [file pcbi.1014392.s003.docx]

**S1 Table. Cohort-level source of label assignment and dataset-specific harmonization rules used to derive Sensitive versus Resistant labels across the nine GEO cohorts.** For cohorts with explicit study-defined binary response groups, those labels were used directly. For longitudinal, treatment-stage, or subset-constructed cohorts, “Sensitive” and “Resistant” denote harmonized operational labels used for binary benchmarking rather than direct single-cell functional viability measurements.

| **Dataset (GEO)** | **Source of label assignment used for harmonization** | **Original study state / subset used in preprocessing** | **Binary outcome definition used in this study** |
| --- | --- | --- | --- |
| GSE111014 | GEO sample metadata and cohort-level response annotation table | Selected pre- and on-treatment samples (d0 and d120/d150, depending on patient availability) were retained, with each sample linked to a patient-level clinical response category | Clinical-response-based labels: samples from patients with PR/CR were mapped to Sensitive, whereas samples from patients with PD were mapped to Resistant; the pre/post timepoint field was retained as metadata but was not itself used as the binary label |
| GSE117872 | GEO sample annotations and study-defined cisplatin-response metadata | Patient-derived OSCC cells from primary and metastatic sites with cisplatin-related states annotated as Sensitive, Resistant, or Drug-holiday | Study-defined response labels: cisplatin-sensitive cells were mapped to Sensitive, cisplatin-resistant cells were mapped to Resistant, and Drug-holiday cells were excluded from the final binary benchmark |
| GSE149214 | GEO sample metadata and preprocessing-defined subset selection | Only untreated Day00b PC9 cells (GSM4494347) and retained Day11 erlotinib-treated PC9 cells (GSM4494349) were used for the final binary comparison | Operational treatment-state labels: Day00b cells were mapped to Sensitive, whereas retained Day11 erlotinib-treated cells were mapped to Resistant |
| GSE149383 (using the time-course subseries GSE134839) | GEO sample titles and erlotinib exposure time-point metadata | PC9 cells were profiled across sequential erlotinib exposure states, including Day 0, Day 1, Day 2, Day 4, Day 9, and Day 11 | Operational time-course labels: untreated/Day 0 cells were used as the Sensitive reference, whereas Day 9 and Day 11 erlotinib-exposed cells were mapped to Resistant |
| GSE152469 | GEO sample metadata and preprocessing-defined longitudinal stage selection | Only GSM4616298 (M0, before treatment) and GSM4616300 (M27, time of progression) were retained for the final binary comparison | Operational longitudinal labels: M0 cells were mapped to Sensitive, and M27 progression-stage cells were mapped to Resistant |
| GSE140440 | GEO series summary, preprocessing notes, and sample-level status table | Docetaxel-treated prostate cancer cell lines DU145 and PC3, each represented by parental and DR states | Study-defined sample-status labels: parental cells were mapped to Sensitive, whereas DR cells were mapped to Resistant |
| GSE131984 (JQ1) | GEO processed metadata and drug-specific sample annotation table | In the filtered SUM159 subset, Pre and DMSO were pre-drug/reference states, whereas JQ1-A and JQ1-B were post-drug JQ1-treated states | Operational treatment labels: Pre + DMSO cells were mapped to Sensitive, and JQ1-A + JQ1-B cells were mapped to Resistant |
| GSE131984 (Paclitaxel) | GEO processed metadata and drug-specific sample annotation table | In the filtered SUM159 subset, Pre and DMSO were reference states, and Tax was the single-agent paclitaxel-treated state | Operational treatment labels: Pre + DMSO cells were mapped to Sensitive, and Tax cells were mapped to Resistant |
| GSE131984 (Palbociclib) | GEO processed metadata and drug-specific sample annotation table | In the filtered SUM159 subset, Pre and DMSO were reference states, and Pal was the single-agent palbociclib-treated state | Operational treatment labels: Pre + DMSO cells were mapped to Sensitive, and Pal cells were mapped to Resistant |
