# Supplementary material for: scRADAR: Dissecting intratumoral drug response heterogeneity at single-cell resolution via mechanism-guided prototype routing
Source: PLoS Comput Biol. 2026 Jun 26;22(6):e1014392. doi: 10.1371/journal.pcbi.1014392 (PMC13309031; doi:10.1371/journal.pcbi.1014392)
Supplement: S2 Table — Full scRADAR was evaluated under four pathway-feature normalization settings using the same model architecture, data-splitting protocol, and threshold-selection procedure. The default setting corresponds to training-cell-fitted z-score standardization, in which scaling parameters were estimated only from the training cells and then applied unchanged to validation and held-out test cells. The other settings were evaluated only for sensitivity analysis and were not used for model selection. Values are shown as mean ± 95% t-interval across cross-validation-derived runs. (DOCX) [file pcbi.1014392.s004.docx]

**S2 Table. Normalization sensitivity analysis of full scRADAR.** Full scRADAR was evaluated under four pathway-feature normalization settings using the same model architecture, data-splitting protocol, and threshold-selection procedure. The default setting corresponds to training-cell-fitted z-score standardization, in which scaling parameters were estimated only from the training cells and then applied unchanged to validation and held-out test cells. The other settings were evaluated only for sensitivity analysis and were not used for model selection. Values are shown as mean ± 95% t-interval across cross-validation-derived runs.

| Normalization setting | AUROC | AUPRC | F1 |
| --- | --- | --- | --- |
| Training-cell-fitted z-score standardization (default) | 0.967_±0.005_ | 0.964_±0.003_ | 0.956_±0.007_ |
| Robust median/IQR | 0.965_±0.005_ | 0.960_±0.006_ | 0.953_±0.007_ |
| Quantile-normal | 0.971_±0.004_ | 0.966_±0.005_ | 0.958_±0.006_ |
| Min-max [0,1] | 0.966_±0.006_ | 0.962_±0.006_ | 0.955_±0.007_ |
