# Supplementary material for: scRADAR: Dissecting intratumoral drug response heterogeneity at single-cell resolution via mechanism-guided prototype routing
Source: PLoS Comput Biol. 2026 Jun 26;22(6):e1014392. doi: 10.1371/journal.pcbi.1014392 (PMC13309031; doi:10.1371/journal.pcbi.1014392)
Supplement: S3 Table — The default full scRADAR setting used Reactome ssGSEA and PROGENy as the dual-view pathway representation. For sensitivity analysis, the pathway representation was modified while the drug fingerprint, FiLM conditioning, prototype-routing module, data-splitting protocol, and threshold-selection procedure were kept unchanged. The ssGSEA-only and PROGENy-only variants removed one pathway view to assess the contribution of each view separately. The Hallmark and Reactome + Hallmark settings were evaluated to test whether performance depended on the selected ssGSEA gene-set collection. These sensitivity analyses were not used for model selection. Values are shown as mean ± 95% t-interval across cross-validation-derived runs. (DOCX) [file pcbi.1014392.s005.docx]

**S3 Table. Pathway-view and pathway-set sensitivity analysis of scRADAR.** The default full scRADAR setting used Reactome ssGSEA and PROGENy as the dual-view pathway representation. For sensitivity analysis, the pathway representation was modified while the drug fingerprint, FiLM conditioning, prototype-routing module, data-splitting protocol, and threshold-selection procedure were kept unchanged. The ssGSEA-only and PROGENy-only variants removed one pathway view to assess the contribution of each view separately. The Hallmark and Reactome + Hallmark settings were evaluated to test whether performance depended on the selected ssGSEA gene-set collection. These sensitivity analyses were not used for model selection. Values are shown as mean ± 95% t-interval across cross-validation-derived runs.

| Variant | Pathway features | AUROC | AUPRC | F1 |
| --- | --- | --- | --- | --- |
| Full scRADAR default | Reactome ssGSEA + PROGENy | 0.967_±0.005_ | 0.964_±0.003_ | 0.956_±0.007_ |
| ssGSEA only | Reactome ssGSEA | 0.943_±0.017_ | 0.934_±0.017_ | 0.929_±0.017_ |
| PROGENy only | PROGENy | 0.937_±0.015_ | 0.926_±0.014_ | 0.923_±0.015_ |
| Hallmark + PROGENy | Hallmark ssGSEA + PROGENy | 0.961_±0.006_ | 0.959_±0.005_ | 0.957_±0.008_ |
| Reactome + Hallmark + PROGENy | Reactome ssGSEA + Hallmark ssGSEA + PROGENy | 0.969_±0.004_ | 0.965_±0.004_ | 0.954_±0.006_ |
