# Supplementary material for: scRADAR: Dissecting intratumoral drug response heterogeneity at single-cell resolution via mechanism-guided prototype routing
Source: PLoS Comput Biol. 2026 Jun 26;22(6):e1014392. doi: 10.1371/journal.pcbi.1014392 (PMC13309031; doi:10.1371/journal.pcbi.1014392)
Supplement: S4 Table — B-cell-specific signatures were evaluated only in the GSE111014 Ibrutinib cohort as a disease-context sensitivity analysis. The default setting used Reactome ssGSEA and PROGENy, whereas the augmented settings added B-cell-specific ssGSEA signatures, with or without Hallmark ssGSEA features. B-cell-specific signatures included CD40 signaling, NF-κB signaling, B-cell receptor signaling, BTK downstream signaling, B-cell activation, apoptosis, antigen presentation, and proliferation-related programs. The full scRADAR architecture, drug fingerprint, FiLM conditioning, prototype-routing module, data-splitting protocol, and threshold-selection procedure were kept unchanged across all settings. Retained total features indicate the final number of pathway-level input features after pathway scoring and feature construction; retained B-cell signatures indicate the number of B-cell-specific signatures included in the feature set. These sensitivity analyses were not used for model selection. Values are shown as mean ± 95% t-interval across cross-validation-derived runs. (DOCX) [file pcbi.1014392.s006.docx]

**S4 Table. GSE111014 B-cell pathway-signature sensitivity analysis.** B-cell-specific signatures were evaluated only in the GSE111014 Ibrutinib cohort as a disease-context sensitivity analysis. The default setting used Reactome ssGSEA and PROGENy, whereas the augmented settings added B-cell-specific ssGSEA signatures, with or without Hallmark ssGSEA features. B-cell-specific signatures included CD40 signaling, NF-κB signaling, B-cell receptor signaling, BTK downstream signaling, B-cell activation, apoptosis, antigen presentation, and proliferation-related programs. The full scRADAR architecture, drug fingerprint, FiLM conditioning, prototype-routing module, data-splitting protocol, and threshold-selection procedure were kept unchanged across all settings. Retained total features indicate the final number of pathway-level input features after pathway scoring and feature construction; retained B-cell signatures indicate the number of B-cell-specific signatures included in the feature set. These sensitivity analyses were not used for model selection. Values are shown as mean ± 95% t-interval across cross-validation-derived runs.

| Dataset | Drug | Pathway/signature setting | Retained total features | Retained B-cell signatures | AUROC | AUPRC | F1 |
| --- | --- | --- | --- | --- | --- | --- | --- |
| GSE111014 | Ibrutinib | Reactome ssGSEA + PROGENy (default) | 404 | 0 | 0.987  ±0.004 | 0.982  ±0.006 | 0.968  ±0.007 |
| GSE111014 | Ibrutinib | Reactome ssGSEA + PROGENy + B-cell-specific ssGSEA signatures | 412 | 8 | 0.986  ±0.005 | 0.985  ±0.004 | 0.970  ±0.005 |
| GSE111014 | Ibrutinib | Reactome ssGSEA + Hallmark ssGSEA + PROGENy + B-cell-specific ssGSEA signatures | 449 | 8 | 0.988  ±0.003 | 0.984  ±0.005 | 0.971  ±0.004 |
