# Supplementary material for: scRADAR: Dissecting intratumoral drug response heterogeneity at single-cell resolution via mechanism-guided prototype routing
Source: PLoS Comput Biol. 2026 Jun 26;22(6):e1014392. doi: 10.1371/journal.pcbi.1014392 (PMC13309031; doi:10.1371/journal.pcbi.1014392)
Supplement: S5 Table — The scGEN-adapted baseline was included as a representative single-cell perturbation-oriented comparator. Because the curated cohorts provide cohort-harmonized binary response-associated labels rather than paired perturbation trajectories for every drug condition, scGEN was adapted as a scGEN-style variational latent representation learner using the same Reactome ssGSEA + PROGENy cellular pathway representation as the base input. The learned latent representation was then used for downstream binary response-phenotype classification under the same target-domain data-splitting and threshold-selection protocol. XGBoost is shown as a strong target-domain supervised comparator, whereas scRADAR represents the full proposed model. Values are shown as mean ± 95% t-interval across cross-validation-derived runs. (DOCX) [file pcbi.1014392.s007.docx]

**S5 Table. scGEN-adapted baseline comparison.** The scGEN-adapted baseline was included as a representative single-cell perturbation-oriented comparator. Because the curated cohorts provide cohort-harmonized binary response-associated labels rather than paired perturbation trajectories for every drug condition, scGEN was adapted as a scGEN-style variational latent representation learner using the same Reactome ssGSEA + PROGENy cellular pathway representation as the base input. The learned latent representation was then used for downstream binary response-phenotype classification under the same target-domain data-splitting and threshold-selection protocol. XGBoost is shown as a strong target-domain supervised comparator, whereas scRADAR represents the full proposed model. Values are shown as mean ± 95% t-interval across cross-validation-derived runs.

| Model | Cellular representation | AUROC | AUPRC | F1 |
| --- | --- | --- | --- | --- |
| scGEN-adapted | Reactome ssGSEA + PROGENy, encoded as a scGEN-style VAE latent | 0.884_±0.022_ | 0.867_±0.026_ | 0.852_±0.024_ |
| XGBoost | Reactome ssGSEA + PROGENy | 0.908_±0.017_ | 0.898_±0.017_ | 0.872_±0.018_ |
| scRADAR  (Ours) | Reactome ssGSEA + PROGENy | 0.967_±0.005_ | 0.964_±0.003_ | 0.956_±0.007_ |
