# Supplementary material for: scRADAR: Dissecting intratumoral drug response heterogeneity at single-cell resolution via mechanism-guided prototype routing
Source: PLoS Comput Biol. 2026 Jun 26;22(6):e1014392. doi: 10.1371/journal.pcbi.1014392 (PMC13309031; doi:10.1371/journal.pcbi.1014392)
Supplement: S6 Table — In each split, two GSE131984 drug-specific subsets were used for training and the remaining drug was held out for testing. No cells from the held-out drug were used for model training, validation, threshold selection, or hyperparameter selection. Cell-only MLP used the shared cellular pathway representation. Concat MLP used the cellular pathway representation concatenated with the mechanism-aware drug fingerprint. Hadamard MLP used an element-wise drug-cell interaction term to evaluate a generic nonlinear drug-cell interaction baseline. Full scRADAR used FiLM-conditioned pathway embeddings followed by sparse prototype routing. Sensitive-label prevalence indicates the fraction of Sensitive-labeled cells in the held-out drug subset. Values are shown as mean ± 95% t-interval across five random validation splits. Macro averages were computed across the three held-out drugs using unrounded held-out-drug means. (DOCX) [file pcbi.1014392.s008.docx]

**S6 Table. Controlled within-study unseen-drug transfer and drug-cell interaction ablation in GSE131984.** In each split, two GSE131984 drug-specific subsets were used for training and the remaining drug was held out for testing. No cells from the held-out drug were used for model training, validation, threshold selection, or hyperparameter selection. Cell-only MLP used the shared cellular pathway representation. Concat MLP used the cellular pathway representation concatenated with the mechanism-aware drug fingerprint. Hadamard MLP used an element-wise drug-cell interaction term to evaluate a generic nonlinear drug-cell interaction baseline. Full scRADAR used FiLM-conditioned pathway embeddings followed by sparse prototype routing. Sensitive-label prevalence indicates the fraction of Sensitive-labeled cells in the held-out drug subset. Values are shown as mean ± 95% t-interval across five random validation splits. Macro averages were computed across the three held-out drugs using unrounded held-out-drug means.

**(A) Held-out-drug performance**

| Held-out drug | Model | Test cells | Sensitive-label prevalence | AUROC | AUPRC | F1 |
| --- | --- | --- | --- | --- | --- | --- |
| JQ1 | Cell-only MLP | 2603 | 0.406 | 0.653_±0.068_ | 0.559_±0.074_ | 0.583_±0.055_ |
| JQ1 | Concat MLP | 2603 | 0.406 | 0.704_±0.042_ | 0.615_±0.038_ | 0.598_±0.041_ |
| JQ1 | Hadamard MLP | 2603 | 0.406 | 0.758_±0.033_ | 0.653_±0.045_ | 0.612_±0.039_ |
| JQ1 | Full scRADAR | 2603 | 0.406 | 0.785_±0.021_ | 0.681_±0.026_ | 0.627_±0.019_ |
| Paclitaxel | Cell-only MLP | 1805 | 0.586 | 0.485_±0.054_ | 0.588_±0.061_ | 0.601_±0.048_ |
| Paclitaxel | Concat MLP | 1805 | 0.586 | 0.626_±0.031_ | 0.638_±0.036_ | 0.694_±0.027_ |
| Paclitaxel | Hadamard MLP | 1805 | 0.586 | 0.554_±0.072_ | 0.591_±0.081_ | 0.641_±0.065_ |
| Paclitaxel | Full scRADAR | 1805 | 0.586 | 0.640_±0.024_ | 0.655_±0.029_ | 0.686_±0.033_ |
| Palbociclib | Cell-only MLP | 1737 | 0.609 | 0.716_±0.025_ | 0.779_±0.022_ | 0.748_±0.028_ |
| Palbociclib | Concat MLP | 1737 | 0.609 | 0.782_±0.018_ | 0.823_±0.015_ | 0.775_±0.017_ |
| Palbociclib | Hadamard MLP | 1737 | 0.609 | 0.815_±0.022_ | 0.856_±0.020_ | 0.796_±0.024_ |
| Palbociclib | Full scRADAR | 1737 | 0.609 | 0.831_±0.011_ | 0.869_±0.013_ | 0.817_±0.014_ |

**(B) Macro-average performance**

| Model | Macro AUROC | Macro AUPRC | Macro F1 |
| --- | --- | --- | --- |
| Cell-only MLP | 0.618_±0.031_ | 0.642_±0.035_ | 0.644_±0.028_ |
| Concat MLP | 0.704_±0.019_ | 0.692_±0.018_ | 0.689_±0.016_ |
| Hadamard MLP | 0.709_±0.027_ | 0.700_±0.032_ | 0.683_±0.026_ |
| Full scRADAR | 0.752_±0.012_ | 0.735_±0.014_ | 0.710_±0.011_ |
