# Supplementary material for: scRADAR: Dissecting intratumoral drug response heterogeneity at single-cell resolution via mechanism-guided prototype routing
Source: PLoS Comput Biol. 2026 Jun 26;22(6):e1014392. doi: 10.1371/journal.pcbi.1014392 (PMC13309031; doi:10.1371/journal.pcbi.1014392)
Supplement: S7 Table — Cluster-level enrichment was evaluated using the cells included in the post hoc Fig 4A–4C analysis by comparing the observed Sensitive-label fraction in each transcriptional cluster with a permutation-derived null distribution that preserved cluster size and cohort-level label composition. Empirical P values were estimated using 5,000 label permutations and adjusted using the Benjamini–Hochberg procedure within each cohort. Clusters were annotated as Sensitive-enriched or Resistant-enriched when the observed Sensitive-label fraction was above or below the cohort-level Sensitive-label fraction, respectively, and the permutation FDR q-value was below 0.05. Clusters with fewer than 20 cells were interpreted cautiously because enrichment estimates are more sensitive to sampling variation. (DOCX) [file pcbi.1014392.s009.docx]

**S7 Table. Cluster-level response-label enrichment analysis for Fig 4A–C.** Cluster-level enrichment was evaluated using the cells included in the post hoc Fig 4A–C analysis by comparing the observed Sensitive-label fraction in each transcriptional cluster with a permutation-derived null distribution that preserved cluster size and cohort-level label composition. Empirical P values were estimated using 5,000 label permutations and adjusted using the Benjamini–Hochberg procedure within each cohort. Clusters were annotated as Sensitive-enriched or Resistant-enriched when the observed Sensitive-label fraction was above or below the cohort-level Sensitive-label fraction, respectively, and the permutation FDR q-value was below 0.05. † indicates clusters with fewer than 20 cells, which were interpreted cautiously because enrichment estimates are more sensitive to sampling variation.

| Panel | Dataset / drug | Cluster | No. of cells | Sensitive-label fraction | Permutation FDR q | Enrichment |
| --- | --- | --- | --- | --- | --- | --- |
| Fig 4A | GSE117872 / Cisplatin | 0 | 180 | 0.494 | <0.001 | Resistant-enriched |
| Fig 4A | GSE117872 / Cisplatin | 1 | 172 | 0.552 | 0.001 | Resistant-enriched |
| Fig 4A | GSE117872 / Cisplatin | 2 | 130 | 0.992 | <0.001 | Sensitive-enriched |
| Fig 4A | GSE117872 / Cisplatin | 3 | 92 | 1.000 | <0.001 | Sensitive-enriched |
| Fig 4A | GSE117872 / Cisplatin | 4 | 91 | 1.000 | <0.001 | Sensitive-enriched |
| Fig 4A | GSE117872 / Cisplatin | 5 | 90 | 0.011 | <0.001 | Resistant-enriched |
| Fig 4A | GSE117872 / Cisplatin | 6 | 85 | 0.000 | <0.001 | Resistant-enriched |
| Fig 4A | GSE117872 / Cisplatin | 7 | 77 | 0.987 | <0.001 | Sensitive-enriched |
| Fig 4A | GSE117872 / Cisplatin | 8 | 75 | 1.000 | <0.001 | Sensitive-enriched |
| Fig 4A | GSE117872 / Cisplatin | 9 | 35 | 1.000 | <0.001 | Sensitive-enriched |
| Fig 4A | GSE117872 / Cisplatin | 10 | 20 | 0.600 | 0.637 | Not significant |
| Fig 4B | GSE149214 / Erlotinib | 0 | 1091 | 0.853 | <0.001 | Sensitive-enriched |
| Fig 4B | GSE149214 / Erlotinib | 1 | 914 | 0.013 | <0.001 | Resistant-enriched |
| Fig 4B | GSE149214 / Erlotinib | 2 | 347 | 0.432 | 0.342 | Not significant |
| Fig 4B | GSE149214 / Erlotinib | 3† | 18 | 0.444 | 1.000 | Not significant |
| Fig 4B | GSE149214 / Erlotinib | 4† | 16 | 0.438 | 1.000 | Not significant |
| Fig 4C | GSE149383 / Erlotinib | 0 | 1087 | 0.855 | <0.001 | Sensitive-enriched |
| Fig 4C | GSE149383 / Erlotinib | 1 | 1068 | 0.027 | <0.001 | Resistant-enriched |
| Fig 4C | GSE149383 / Erlotinib | 2 | 355 | 0.451 | 0.832 | Not significant |
| Fig 4C | GSE149383 / Erlotinib | 3† | 19 | 0.368 | 0.832 | Not significant |
| Fig 4C | GSE149383 / Erlotinib | 4† | 19 | 0.474 | 0.832 | Not significant |
| Fig 4C | GSE149383 / Erlotinib | 5† | 18 | 0.333 | 0.832 | Not significant |
