# Supplementary material for: scRADAR: Dissecting intratumoral drug response heterogeneity at single-cell resolution via mechanism-guided prototype routing
Source: PLoS Comput Biol. 2026 Jun 26;22(6):e1014392. doi: 10.1371/journal.pcbi.1014392 (PMC13309031; doi:10.1371/journal.pcbi.1014392)
Supplement: S8 Table — Palbociclib Cluster 0 was characterized using held-out cells assigned to this transcriptional neighborhood. AUROC and AUPRC were computed from predicted Sensitive probabilities and cohort-harmonized response-associated labels. Because Cluster 0 was Resistant-labeled-cell-enriched and contained only 16 Sensitive-labeled cells, AUPRC was interpreted relative to the local Sensitive-label prevalence baseline. The Mann–Whitney test compared predicted Sensitive probabilities between Sensitive-labeled and Resistant-labeled cells within this cluster. Reactome pathway deviations were computed from model-derived pathway activity profiles. These analyses were used only for post hoc characterization and were not used for model training, threshold selection, or prospective prediction. (DOCX) [file pcbi.1014392.s010.docx]

**S8 Table. Post hoc characterization of Palbociclib Cluster 0.** Palbociclib Cluster 0 was characterized using held-out cells assigned to this transcriptional neighborhood. AUROC and AUPRC were computed from predicted Sensitive probabilities and cohort-harmonized response-associated labels. Because Cluster 0 was Resistant-labeled-cell-enriched and contained only 16 Sensitive-labeled cells, AUPRC was interpreted relative to the local Sensitive-label prevalence baseline. The Mann–Whitney test compared predicted Sensitive probabilities between Sensitive-labeled and Resistant-labeled cells within this cluster. Reactome pathway deviations were computed from model-derived pathway activity profiles. These analyses were used only for post hoc characterization and were not used for model training, threshold selection, or prospective prediction.

| Analysis | Result | Interpretation |
| --- | --- | --- |
| Cluster composition | 16 Sensitive-labeled / 86 Resistant-labeled cells | Resistant-labeled-cell-enriched neighborhood |
| Local AUROC | 0.948 | Strong local score-label separation |
| Local AUPRC | 0.703; prevalence baseline = 0.157 | 4.5-fold above the local prevalence baseline |
| Median probability, Sensitive-labeled cells | 0.940 | Sensitive-labeled cells received high predicted Sensitive probabilities |
| Median probability, Resistant-labeled cells | 0.010 | Resistant-labeled cells received low predicted Sensitive probabilities |
| Median probability difference | 0.930 | Clear separation of local score distributions |
| Mann–Whitney test | P = 1.40 × 10^-8^ | Significant separation of predicted Sensitive probabilities |
| Top model-derived pathway deviations | Cell-cycle and mitotic programs | Cell-cycle-related transcriptional state |
