# Supplementary material for: scRADAR: Dissecting intratumoral drug response heterogeneity at single-cell resolution via mechanism-guided prototype routing
Source: PLoS Comput Biol. 2026 Jun 26;22(6):e1014392. doi: 10.1371/journal.pcbi.1014392 (PMC13309031; doi:10.1371/journal.pcbi.1014392)
Supplement: S9 Table — Representative prototypes were characterized after model training using cells with the highest routing weights to each prototype. Response-label enrichment was evaluated by testing whether the top-routed cells were enriched for Sensitive-labeled or Resistant-labeled cells relative to the corresponding cohort background. Transcriptional-cluster enrichment was evaluated by testing whether the same top-routed cells were overrepresented in specific transcriptional clusters. FDR q-values were obtained after multiple-testing correction. Prototype labels such as Sensitive-labeled enriched or Resistant-labeled enriched were assigned only as post hoc summaries of enriched routed cells; the prototypes were not predefined as Sensitive or Resistant classes during model training. These analyses were used only for interpretability assessment and were not used for model training, model selection, threshold selection, or prospective prediction. (DOCX) [file pcbi.1014392.s011.docx]

**S9 Table. Representative post hoc characterization of learned prototypes.** Representative prototypes were characterized after model training using cells with the highest routing weights to each prototype. Response-label enrichment was evaluated by testing whether the top-routed cells were enriched for Sensitive-labeled or Resistant-labeled cells relative to the corresponding cohort background. Transcriptional-cluster enrichment was evaluated by testing whether the same top-routed cells were overrepresented in specific transcriptional clusters. FDR q-values were obtained after multiple-testing correction. Prototype labels such as Sensitive-labeled enriched or Resistant-labeled enriched were assigned only as post hoc summaries of enriched routed cells; the prototypes were not predefined as Sensitive or Resistant classes during model training. These analyses were used only for interpretability assessment and were not used for model training, model selection, threshold selection, or prospective prediction.

| Dataset / drug | Prototype | No. of top-routed cells | Response-label enrichment | Label FDR q | Enriched transcriptional cluster | Cluster FDR q |
| --- | --- | --- | --- | --- | --- | --- |
| GSE131984 / Palbociclib | P4 | 20 | Resistant-labeled enriched | 1.5 × 10^-8^ | Cluster 0 | 1.3 × 10^-10^ |
| GSE131984 / Palbociclib | P6 | 20 | Sensitive-labeled enriched | 9.9 × 10^-5^ | Cluster 2 | 1.5 × 10^-12^ |
| GSE131984 / JQ1 | P4 | 27 | Sensitive-labeled enriched | 6.5 × 10^-11^ | Cluster 3 | 5.7 × 10^-12^ |
| GSE131984 / JQ1 | P12 | 27 | Resistant-labeled enriched | 1.1 × 10^-6^ | Cluster 1 | 1.5 × 10^-14^ |
| GSE149214 / Erlotinib | P7 | 24 | Sensitive-labeled enriched | 6.0 × 10^-8^ | Cluster 0 | 7.6 × 10^-8^ |
| GSE149214 / Erlotinib | P5 | 24 | Resistant-labeled enriched | 1.0 × 10^-6^ | Cluster 2 | 2.0 × 10^-20^ |
| GSE149383 / Erlotinib | P6 | 26 | Sensitive-labeled enriched | 3.2 × 10^-9^ | Cluster 0 | 1.1 × 10^-9^ |
| GSE149383 / Erlotinib | P11 | 26 | Resistant-labeled enriched | 5.6 × 10^-7^ | Cluster 1 | 1.2 × 10^-9^ |
| GSE111014 / Ibrutinib | P9 | 100 | Sensitive-labeled enriched | 1.7 × 10^-31^ | Cluster 6 | 7.0 × 10^-122^ |
| GSE111014 / Ibrutinib | P12 | 100 | Resistant-labeled enriched | 6.2 × 10^-30^ | Cluster 1 | 8.4 × 10^-84^ |
| GSE117872 / Cisplatin | P12 | 20 | Resistant-labeled enriched | 7.0 × 10^-10^ | Cluster 0 | 8.2 × 10^-6^ |
| GSE117872 / Cisplatin | P8 | 20 | Sensitive-labeled enriched | 4.4 × 10^-4^ | Cluster 2 | 3.6 × 10^-20^ |
